# Item-Level Analysis of Category Fluency Test Performance: A Systematic Review and Meta-Analysis of Studies of Normal and Neurologically Abnormal Ageing

**DOI:** 10.1007/s11065-024-09657-z

**Published:** 2025-01-22

**Authors:** Matteo De Marco, Laura M. Wright, Elena Makovac

**Affiliations:** 1 Department of Psychology, College of Health, Medicine and Life Sciences, Brunel University of London, Kingston Lane, Uxbridge, Middlesex UB8 3PH UK; 2 Translational and Clinical Research Institute, Newcastle University, Newcastle-Upon-Tyne, NE1 7RU UK; 3 Department of Neuroimaging, Institute of Psychology, Kings College London, Psychiatry & Neuroscience, London, WC2R 2LS UK

**Keywords:** Semantic fluency, Semantic complexity, Item-based, Dementia, Qualitative scoring, Semantic memory

## Abstract

While Category Fluency (CF) is widely used to help profile semantic memory, item-level scoring (ILS) approaches to this test have been proposed to obtain indices that are less influenced by non-semantic supportive functions. We systematically reviewed the literature to test the hypotheses that (1) compared with healthy adults, individuals with a clinical diagnosis suggestive of neurodegeneration generate words of lower semantic complexity; (2) compared with young adults, older adults generate words of higher semantic complexity. We searched six databases (date of search: 8 December 2023) for studies that relied on CF and ILS methods, in normal ageing and in age-associated neurodegeneration. Thirty-four studies were shortlisted: 27 on neurodegenerative conditions; 7 on normal ageing. Risk of bias was evaluated via a published checklist. Data were presented via qualitative synthesis. Most studies reported words of lower semantic complexity in relation to at least one item-level feature in individuals with mild cognitive impairment (MCI), Alzheimer’s dementia (AD), and other neurodegenerative diseases. *Post-ho*c meta-analyses focussing on the MCI/AD continuum confirmed an effect on words’ frequency (385 MCI/AD individuals and 350 controls; *Hedges’s G* = 0.59) and age-of-acquisition (193 MCI/AD individuals and 161 controls; *Hedges’s G* =  − 1.51). Studies on normal ageing, conversely, failed to demonstrate any overall effect. Most studies on MCI and AD have not relied on neurobiological diagnostic criteria. Moreover, only a small number of studies analysed ILS controlling for quantitative CF performance. Despite these two limitations, this study suggests that ILS can contribute to an in-depth characterisation of semantic memory in neurological ageing.

**Supplementary Information:**

The online version contains supplementary material available at 10.1007/s11065-024-09657-z.

## Introduction

In its original formulation, semantic memory (SM) was defined as the “organized knowledge a person possesses about words and other verbal symbols, their meaning and referents, about relations among them, and about rules, formulas and algorithms for the manipulation of these symbols, concepts and relations” (Tulving, 1972). The current view of semantic cognition holds on to the idea of a multi-componential function. It is, in fact, based on the wealth of information accumulated by a person during the course of their life (i.e. semantic knowledge), but it also includes a set of processes that allows us to use this knowledge flexibly, i.e. semantic control (Lambon Ralph et al., 2017). A third set of aspects, finally, plays a central role when semantic knowledge and semantic control are functional to memory processes: those of encoding and retrieval.

While a clear theoretical framework that recognises the distinct components of semantic cognition is important from an academic standpoint, it is also informative to elucidate the mechanisms that define the trajectories of decline and retained competence in normal ageing and in the population of individuals who suffer from neurodegenerative conditions. A strong body of evidence indicates that semantic knowledge consolidates and even improves with normal ageing (Grady, 2012; Nilsson, 2003; Park et al., 2002; Rönnlund et al., 2005; Verhaeghen, 2003), while semantic control appears instead to decline in older adults (Ambrosini et al., 2023; Hoffman, 2019). In a neurodegenerative condition such as Alzheimer’s disease (AD), on the other hand, a quantifiable decline is seen in relation to both semantic control *and* semantic knowledge (Garrard et al., 2005; Laatu et al., 1997; Mascali et al., 2018).

Although influenced by diverse functions, the Category Fluency test (CFT) is an instrument that has been long used to assess SM. It is a brief task in which the testee is asked to name as many words as possible that belong to a certain category. This is typically carried out within a time constraint (usually 1 min). The number of correct entries is then counted, and this count is extracted as a test score. Box 1 includes a real-world example of performance (plus examples of incorrect entries and scoring rules) shown by a young adult, in three distinct categories. The CFT was designed in the 1940s (Bousfield & Sedgewick, 1944) and, over the years, has been used to assess a wide range of clinical conditions (as documented by meta-analytical publications), including amnestic Mild Cognitive Impairment (MCI—Sharma & Malek-Ahmedi, 2023), AD (Henry et al., 2004; Olmos-Villaseñor et al., 2023), Parkinson’s disease (PD—Henry & Crawford, 2004), epilepsy (Metternich et al., 2014), depression (Henry & Crawford, 2005), schizophrenia (Bokat & Goldberg, 2003), and bipolar disorder (Raucher-Chéné et al., 2017).

**Box 1** Example of CFT performance and scoring on three categories (1 min each)

**Table:** 

Animals	Fruits	Musical instruments
a01: DOG a02: CAT a03: COW a04: PIG a05: BULL a06: HORSE a07: BIRD a08: ELEPHANT a09: GIRAFFE a10: RHINO a11: **OWL** a12: SQUIRREL a13: WHALE a14: FISH a15: **COD** a16: DOLPHIN a17: ANT a18: BEE a19: FLY a20: BUTTERFLY a21: GOAT a22: **HORSE** *n* = 19	f01: APPLE f02: ORANGE f03: BANANA f04: PEAR f05: PLUM f06: PEACH f07: APRICOT f08: AVOCADO f09: **TOMATO** f10: PINEAPPLE f11: RASPBERRY f12: GOOSEBERRY f13: STRAWBERRY f14: BLACKBERRY f15: BLACKCURRANT f16: RHUBARB f17: LEMON f18: LIME f19: GRAPE *n* = 18	mi01: PIANO mi02: DRUM mi03: PICCOLO mi04: VIOLIN mi05: VIOLA mi06: CELLO mi07: DOUBLE BASS mi08: GUITAR mi09: **ELECTRIC GUITAR** mi10: TIN WHISTLE mi11: ACCORDION mi12: SHAKERS mi13: MARACAS mi14: RECORDER mi15: CYMBALS mi16: TRIANGLE *n* = 15
Total count = 52		

Performance of a 21-year-old right-handed, male, native English speaker on three distinct categories. A set of rules is typically applied to identify the two “recognised” classes of CFT errors: *perseverations* and *intrusions*. These might be based on arbitrary principles, for instance, in the above performance, while *a22* is an exact repetition (i.e. *perseveration*) of a word previously generated (i.e. *a06*), *a11*, *a15*, and *mi09* might be also counted as *perseverations* as they are subordinate exemplars of words previously generated (i.e. *a07*, *a14*, and *mi08*, respectively). In this specific case, superordinate or subordinate words are arbitrarily accepted as correct based on which one was named first within the list. In this example, *f09* was marked (again, arbitrarily) as an intrusion. The total count (and, thus, the CFT score) in a three-category version of the test is the arithmetical sum of the three sub-counts

Although various neuropsychological tools exist to assess semantic memory (e.g. Pyramids and Palm Trees test, Delayed Matching-to-Sample 48 test, Wechsler Adult Intelligence Scale Similarities test), the CFT offers a number of advantages. From a methodological and procedural viewpoint, it is easy to administer (i.e. the tester does not have to undergo extensive training) and to carry out (even for individuals with a severe clinical profile), and it can be easily transposed to any linguistic and cultural setting without the need for validation studies. Moreover, as it is a test of free recall (Gruenewald & Lockhead, 1980), it is characterised by a particularly high ecological validity, as free recall is the form of memory retrieval that is most distinctively at the basis of daily-life memory demands (Craik, 1983).

Although these are notable advantages, a major limitation is recognised. The count of correct entries (Box 1) is not exclusively reflective of SM abilities. A large number of studies indicate that other functions such as executive functioning, attention, and speed of processing also play a major role in the score’s construct validity (Aita et al., 2019; Elgamal et al., 2011; Gibbons et al., 2012; Shao et al., 2014). This is of particular relevance to those neurological conditions that show SM decline at their earliest stages, such as AD (Venneri et al., 2016, 2018) and the semantic variant of Primary Progressive Aphasia (PPA—Mendez et al., 2020). In these conditions, a precise characterisation of SM free recall performance could help define better diagnostic algorithms and, potentially, anticipate the time of diagnosis at the preclinical stage, if the test is particularly sensitive to SM decline, and if its underlying validity is not significantly influenced by other, non-SM abilities, which might act as ancillary supportive functions. Both AD (Garrard et al., 2005; Laatu et al., 1997; Mascali et al., 2018) and semantic PPA (Borghesani et al., 2021; Roncero et al., 2020), in fact, are characterised by semantic-knowledge *and* semantic-control degradation.

In response to this limitation, and in the attempt to maximise the informativity of CFT performance, a number of studies have introduced and developed a novel approach to the methods of scoring. This approach is known as *item-level*: entries are individually scored to quantify their “semantic difficulty”, under the assumption that a better-preserved SM would enable an individual to recall more difficult entries (De Marco et al., 2023a). The use of the word “difficulty” derives from the concept of “item difficulty”: “a psychometric property that measures the ease of a test item” (McMillen et al., 2023). Descriptors such as frequency and age of acquisition (i.e. see Box 2 for an extensive list and for the operational definitions included in this systematic review) are considered an expression of item difficulty because they are linked to how efficiently the item is processed, as it is the case, for instance, for words acquired earlier in life (Brysbaert & Biemiller, 2017), and for more concrete words as opposed to more abstract words (Brysbaert et al., 2014). Semantic difficulty has been operationalised in a large number of ways (i.e. see Box 2), in the attempt of characterising a range of “nuances” that might facilitate SM retrieval. The rationale whereby item-level scoring would be less influenced by non-SM abilities lies in the fact that functions such as working memory (Rosen & Engle, 1997) and speed of processing (Elgamal et al., 2011) support control processes (for instance, by allowing a faster search and efficient shifting between subcategories) but would not specifically confer an advantage in retrieving richer semantic information.

**Box 2** Definitions of item-level semantic and non-semantic/relational features

**Table:** 

Feature	Definition*	Example of normative data (where relevant) or reference study	Direction of difficulty (i.e. harder > easier)
a) Semantic item-level features			
Typicality	Numerical index of how prototypical an entry is of the category it is part of	Quaranta et al., 2016	Less typical > more typical
Age of acquisition	Age (in years) at which the entry is learnt	Kuperman et al., 2012	Acquired later > acquired earlier
Frequency	Numerical index of how commonly used a word is	van Heuven et al., 2014	Less frequently used > more frequently used
Prevalence	Proportion of individuals within a cohort who know and recognise the word	Brysbaert et al., 2019	Less prevalent > more prevalent
Recognition time	Average response time taken to identify the entry as a word (also known as “response time")	Mandera et al., 2020	Recognised more slowly > recognised more quickly
Valence	The degree of pleasantness conveyed by the word	Warriner et al., 2013	Less pleasant > more pleasant
Dominance	The degree of perceived control towards the referent of the word	Warriner et al., 2013	Less dominant > more dominant
Arousal	The strength of the emotion conveyed by the word	Warriner et al., 2013	Triggering weaker arousal > triggering stronger arousal^†^
Body-object/sensorimotor interaction	The potential for sensory and motor interaction evoked by the word	Lynott et al., 2020	Evoking weaker sensorimotor strength > evoking stronger sensorimotor strength
Manipulability	The degree to which a word evokes an action pertinent to its recognition	Moreno-Martínez et al., 2014	Less manipulable > more manipulable
Concreteness	The degree to which the word’s referent is a perceptible entity	Brysbaert et al., 2014	More abstract > more concrete
Imageability	The effort of generating a mental image of the word’s referent	Scott et al., 2019	Harder to imagine > easier to imagine
Familiarity	The degree to which the referent(s) of a word is within one’s realm of experience	Scott et al., 2019	Less familiar > more familiar
Semantic diversity	The variability in meaning of a word that is dictated by the various contexts in which it is used	Hoffman et al., 2013	Words with smaller meaning-related variability > words with larger meaning-related variability
Relative occurrence^††^	The proportion of times across the sample/cohort the entry is generated (i.e. as part of the study itself)	N/A	Occurring less often > occurring more often
b) Non-semantic item-level features			
Graphemic length	The number of graphemes used to write the word	N/A	Words with more graphemes > words with fewer graphemes
Phonemic length	The number of phonemes at the basis of the word when it is pronounced	N/A	Words with more phonemes > words with fewer phonemes
Syllabic length	The number of syllables at the basis of the word when it is pronounced	N/A	Words with more syllables > words with fewer syllables
Consonant-to-vowel ratio	Ratio between number of consonants and total number of graphemes/phonemes the entry is composed by	Dufau et al., 2015	Words with larger ratios > words with smaller ratios^†^
Phonological complexity	Pronunciation complexity of consonant clusters	Riley & Thompson, 2015	Phonologically more complex words > phonologically less complex words
c) Relational (item-to-item) features			
Semantic association/semantic neighbourhood (density) / semantic pairwise similarity	Algorithm-based quantification of words co-occurring with a target entry based on a large normative corpus of textual documents	Günther et al., 2015	Words with larger semantic neighbourhood > words with smaller semantic neighbourhood
(“In-list”/dictionary) orthographic Levenshtein distance/orthographic neighbourhood density/one-grapheme orthographic similarity	A range of lexical indices that are based on the differences in the number of *graphemes* between the target entry and other entries of the dictionary, or of the list of words generated as part of the test, e.g • the number of entries differing by one grapheme from the target entry the average number of graphemes that characterise the lexical distance between the target entry and other entries	Yarkoni et al., 2008	Words with poorer orthographic neighbourhood > words with richer orthographic neighbourhood
Phonological Levenshtein distance/phonological neighbourhood density/one-phoneme phonological similarity	A range of lexical indices that are based on the differences in the number of *phonemes* between the target entry and other entries of the dictionary, or of the list of words generated as part of the test, e.g • the number of entries differing by one phoneme from the target entry • the average number of phonemes that characterise the lexical distance between the target entry and other entries	Vitevitch, 2007	Words with poorer phonological neighbourhood > words with richer phonological neighbourhood
Nodal granularity	Within WordNet (a network representation of the entire lexicon), the number of nodes between an entry and its related “entity of reference” (e.g. “flower” to “rose”)	Sanz et al., 2022	Words with larger granularity > words with smaller granularity

The features listed in (a) are those included in the search of the systematic review (Box 3), with the exception of “relative occurrence”, which is a term introduced in this review to indicate the relative (i.e. sample/cohort specific) proportion of participants who generated the word. Features listed in (b) and (c) were not included in the search, but were nonetheless scored in the studies shortlisted. These are listed here only to provide a definition and facilitate the consultation of Tables 1 and 2

^*^The definitions included in this table and associated with the semantic features refer to studies that have investigated the written form of the words

^†^This directionality is hypothetical. Word arousal, in fact, appears to remain stable throughout the 1-min test performance (despite difficulty typically increases as more words are generated), as demonstrated by a non-significant *z*-converted correlation coefficient between arousal and serial recall order (De Marco & Venneri, 2022)

^††^Although this exact label was not used in the reviewed literature, “relative occurrence” identifies how common entries are in relation to the recruited sample/cohort and not in relation to a set of published norms

Aside from age, a number of inter-individuals and methodological variables are likely to influence the processing of semantic difficulty. Two of these are of particular relevance to clinical settings: cognitive reserve and the number of CFT categories. Cognitive reserve refers to the neurofunctional processes deployed to cope with pathology or damage (Stern et al., 2020). Since semantic processing is supported by a wide network of cortical regions (Binder et al., 2009; Huth et al., 2016), it is reasonable to expect that the ability to elaborate difficult semantic items would be associated with proxies of cognitive reserve, such as years of education. This is confirmed by evidence collected in a large sample of individuals with MCI or AD: those with higher educational attainment performed better on tests characterised by high semantic demands (Darby et al., 2017). Aside from its influence on semantic processing abilities, educational attainment might also be an indicator of the amount of semantic knowledge an individual has been exposed to, with more years spent in education resulting in more knowledge (and, thus, more words) having been encoded. The number of CFT categories is another aspect that deserves attention since, often, "animals" is the only category that is administered (as is the case for the Consortium to Establish a Registry for Alzheimer’s Disease – CERAD, and the “Addenbrooke’s Cognitive Examination III” – ACE III batteries), while other times two or three categories are used (the cognitive battery of the “National Alzheimer’s Coordinating Center” initiative includes "animals" and "vegetables", for instance). If the testee is capable of retrieving semantically difficult items, they will be able to do so across multiple categories, and this may exacerbate the discrepancy between low-performing and high-performing participants. A further aspect that may play a role is the number of CFT words. In fact, an excellent performance may include a large number of semantically difficult items (which would have a positive effect on item-level scores) or may instead largely consist of semantically simple items (which would instead dilute item-level scores).

While item-level approaches have been studied over the years, the literature on the topic is quite scattered and methodologically heterogeneous. We thus designed a systematic review to characterise item-level CFT metrics in normal and neurologically abnormal ageing and provide at the same time a framework of reference for researchers interested in this area of study. Specifically, we wanted to understand whether item-level scores differ (1) between young and older adults and (2) between normal adults and individuals with a condition suggestive of neurodegeneration. As semantic knowledge consolidates with age, we hypothesised that older adults would be able to generate more complex words than young adults. We also hypothesised that normal controls would be able to generate more complex words than individuals with a neurodegenerative condition, although this would emerge more clearly when conditions affecting SM are analysed (i.e. amnestic MCI, AD, and the semantic variant of PPA).

Since we anticipated that item-level scoring methodologies would show a heterogeneous pattern across studies, we deferred possible meta-analyses to *post-hoc* procedures.

## Materials and Methods

### Initial Literature Search

The literature search on the basis of this systematic review was carried out on 8 December 2023. A multi-componential search string was defined to shortlist and identify manuscripts eligible for inclusion as per the study hypothesis. This was aligned with the “PICO” framework (Schardt et al., 2007) and was based on three thematic components: (1) the CFT; (2) the neurological mechanisms/conditions of interest; (3) the set of item-level features used to quantify semantic complexity of individual words. The exact search terms are indicated in Box 3. Terms were searched in the title, keywords, and abstract sections of manuscripts. The search was conducted without any publication-date constraints.

**Box 3** Combination of terms used in the search

**Table:** 

		Approach-related terms		Condition-related terms
“fluency”	AND	“item-level” OR “item-based” OR “typicality” OR “age of acquisition” OR “frequency” OR “recognition time” OR “valence” OR “dominance” OR “body-object interaction” OR “sensorimotor interaction” OR “manipulability” OR “concreteness” OR “affective ratings” OR “arousal” OR “imageability” OR “familiarity” OR “semantic diversity”	AND	“Alzheimer*” OR “dement*” OR “mild cognitive impairment” OR “MCI” OR “vascular” OR “cerebrovascular” OR “cerebro-vascular” OR “frontotemporal” OR “fronto-temporal” OR “FTD” OR “FTLD” OR “Lewy” OR “Parkinson*” OR “semantic dementia” OR “progressive aphasia*” OR “posterior cortical atrophy” OR “amnestic impairment” OR “neurocognitive disorder*” OR “neurodegenerati*” OR “neurological” OR “older” OR “aging” OR “ageing” OR “senior*” OR “elder*”

The list of item-level features that was included was informed by the existence of published normative data. No non-semantic properties such as orthographic or phonological Levenshtein distances or graphemic/syllabic length were included in this search. When included in an eligible study, however, these features were discussed in the qualitative synthesis. Similarly, although the focus was not on Letter Fluency, procedures that calculated composite features from both Category Fluency and Letter Fluency or analysed task interaction effects were included. The exclusion of Letter Fluency Test performance from this systematic review is motivated by methodological, theory- and data-driven aspects. While semantic processing is necessary during CFT performance, it has to be suppressed during Letter Fluency, in order to rely on other strategies of word retrieval (Shao et al., 2014). As a result, task-related neural resources (Biesbroek et al., 2016; Meinzer et al., 2009; Vonk et al., 2019a) and the numerical distribution of item-level features (Gonzalez-Recober et al., 2023) differ significantly between the two tests. Moreover, Letter Fluency performance can be characterised by “phonemic clusters”, i.e. sequences of words that are either homophones or differ by one single vowel sound (Kosmidis et al., 2004), semantic ambiguities (e.g. PITCH as “tar-like substance” vs. PITCH as “musical tone”), and part-of-speech ambiguities (e.g. PITCH as a noun vs. PITCH intended as a verb, as “to throw”), all phenomena that do not distinctively characterise CFT performance (please note that ambiguities are also difficult to score via an item-level approach since the vast majority of normative data do not differentiate between two different meanings or parts of speech). Finally, while it is possible that semantic difficulties might have an impact on the type of words that are generated as part of Letter Fluency performance (see, for instance, Park et al., 2022, for a study investigating the semantic properties of Letter Fluency performance), it is also fair to acknowledge that semantic activation is not a core demand of this task.

A major approach to CFT scoring that was not considered is that based on word clusters. Various methodologies have been proposed to assess “clustering” and “cluster switching” during CFT performance. Although the classic view is that, of the two measures, clustering depends on semantic categorisation abilities (Troyer, 2000), evidence collected in healthy adults shows that it is also significantly influenced by executive functioning (Fong et al., 2020; Unsworth et al., 2011), making it thus less relevant to this systematic review.

The literature search was carried out to cover experimental as well as clinical areas of research, and, to this end, the following databases were queried: “Web of Knowledge”, “MEDLINE”, “CINAHL Plus”, “APA PsycArticles”, “APA PsycINFO”, and “Academic Search Complete”, via bibliographical access to “Web of Science”, “Pubmed”, “Ebsco Host”, and “Ovid”.

### Study Identification and Selection

The output of each of the four bibliographical searches was initially cross-examined to identify duplicate publications. The resulting, duplicate-free list was screened to discard: (1) publications not in English, (2) non-full-length publications (e.g. conference abstracts), and (3) publications referring to thematic areas different from that addressed in this systematic review (i.e. studies that did not focus on the outcome of interest). The word “fluency”, in fact, is also used by clinicians and researchers to indicate other linguistic (e.g. “speech fluency”, “reading fluency”) and non-linguistic (e.g. “perceptual fluency”, “motor fluency”) abilities. At this stage, publications were also discarded if Phonemic/Letter Fluency was the only test investigated, or if CFT was investigated, but the aspects defined by the item-level search term (i.e. the *approach-related terms* listed in Box 3) referred to concepts other than semantic difficulty of CFT entries (e.g. “age of acquisition” indicating the acquisition of a second language, “dominance” related to hemispheric dominance, or “frequency” referring to electrophysiological oscillations).

The full-text of all candidate studies shortlisted at the end of the selection process was independently consulted and assessed for eligibility by all co-authors. All eligible studies were then included in the qualitative synthesis. This was subdivided into two sections: (1) studies focussing on the effect of neurodegenerative conditions and (2) studies focussing on the effect of ageing.

## Results

### Study Shortlisting

**Fig. 1:**
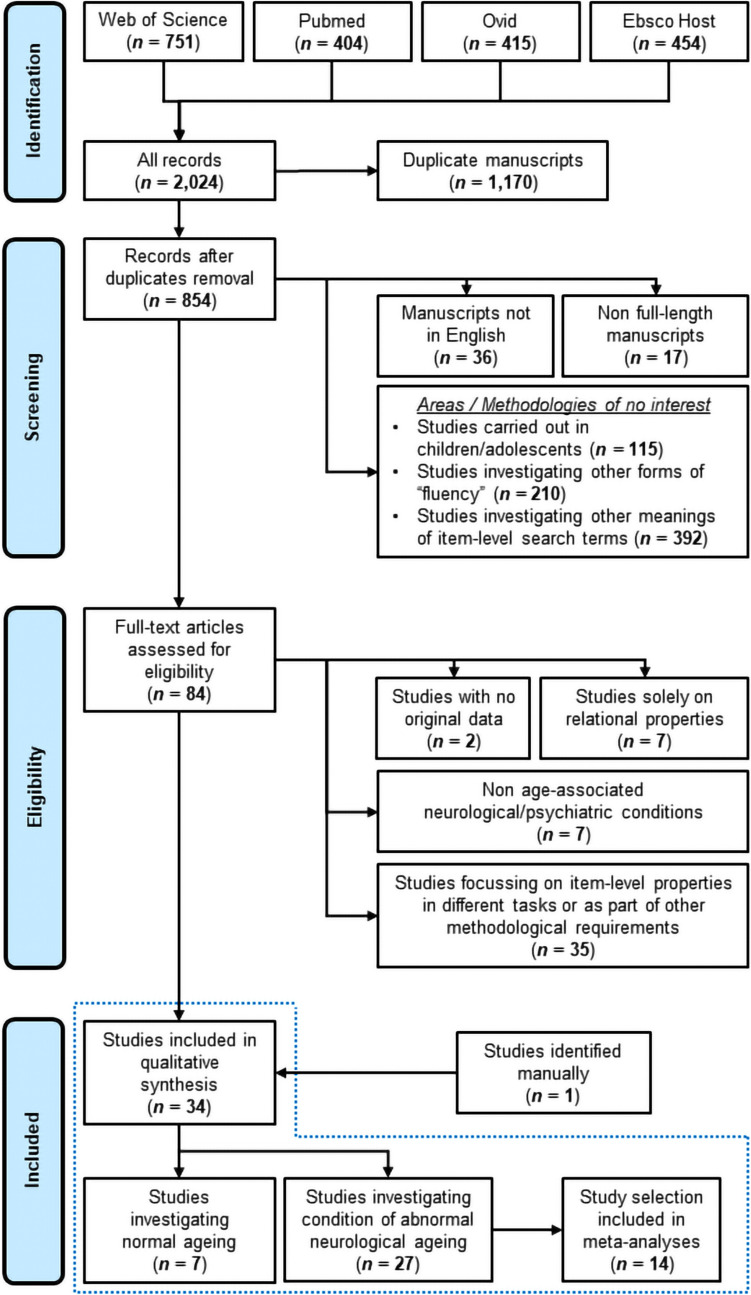
Literature search flowchart

The process of study identification, screening, and assessment for eligibility is illustrated in Fig. 1 and was carried out by the first author. A total of 854 unique entries emerged from the search. Upon the application of the three exclusion criteria described in the “Study Identification and Selection” section, 84 were retained to be assessed for eligibility. Of the discarded manuscripts, 210 studied other forms of fluency, 392 investigated concepts defined by the same *approach-related terms* as those listed in Box 3, but different from those of interest, and a total of 115 were studies carried out in samples of children and adolescents (and were thus easily identified at this stage). The full-text of the remaining 84 manuscripts was accessed to identify those thematically aligned with the hypotheses. Two of these did not include any original data and were not further considered (i.e. De Marco et al., 2023a, b; Venneri et al., 2018). A total of 35 additional studies were discarded as item-level properties were investigated as part of other neuropsychological tasks or to serve non-relevant methodological purposes (e.g. Lam & Marquardt, 2020; Taler & Johns, 2022). Seven additional studies only explored the relational properties of words to calculate performance metrics such as clustering and switching but did not focus on the degree of complexity of retrieved words. Finally, 7 studies were excluded as they investigated item-level scores in other, non-age-associated neurological and psychiatric conditions (e.g. HIV, schizophrenia, autism). The remaining 33 manuscripts were included in the qualitative synthesis. The 33 lists of references were thoroughly checked to identify additional eligible manuscripts. One study was identified at this stage, bringing the total to 34 (27 investigating neurodegenerative conditions and 7 investigating normal ageing). When manuscripts included more sub-studies, those that were eligible were independently entered into the qualitative synthesis. To describe the methodological quality of these studies, a modified version of the checklist by Downs and Black (1998) was compiled by the first author. As carried out elsewhere (Talbot et al., 2024), only the points relevant to observational/quasi-experimental studies were included. These are reported in Table 1. Quality levels ranged between low, to moderate, to excellent (2, 15, and 21 studies/sub-studies, respectively). All studies were approved by an appropriate institutional ethics panel and reported to have been carried out in compliance with The Code of Ethics of the World Medical Association (Declaration of Helsinki). The review was not registered, and no protocol was prepared beforehand.

**Table 1:** Methodological quality assessments of studies included in the systematic review

Study	Reporting							External validity		Internal validity							Quality
	Q1	Q2	Q3	Q5	Q6	Q7	Q10	Q11	Q12	Q16	Q17	Q18	Q20	Q21	Q22	Q25	
*Studies that focussed on neurodegenerative conditions*																	
Beber et al., 2015	1	0	1	2	1	0	1	UTD	0	1	N/A	1	1	N/A	UTD	1	62.50%
Binetti et al., 1995	1	1	1	2	0	1	0	1	0	1	N/A	1	1	N/A	UTD	0	62.50%
Ferrante et al., 2024	1	1	1	2	1	1	1	UTD	0	1	N/A	1	1	1	1	1	87.50%
Forbes-McKay et al., 2005	1	1	1	1	1	1	0	UTD	0	1	N/A	1	1	UTD	UTD	1	62.50%
Henderson et al., 2023	1	N/A	1	1	1	1	1	UTD	0	1	N/A	1	1	UTD	UTD	0	60.00%
Herrera et al., 2012	1	1	1	2	1	1	0	UTD	0	1	N/A	0	1	UTD	UTD	0	56.25%
Hough & Givens, 2004	1	0	1	2	1	0	0	UTD	0	0	N/A	1	1	UTD	UTD	0	43.75%
Jiskoot et al., 2023	1	1	1	2	1	1	1	UTD	0	1	1	1	1	1	1	0	82.35%
Marczinski & Kertesz, 2006	1	1	1	2	1	1	1	UTD	0	1	N/A	1	1	1	1	0	75.00%
Moreno-Martínez & Montoro, 2010 (cross-sectional findings)	1	1	1	2	1	0	1	UTD	0	1	N/A	1	1	UTD	UTD	0	62.50%
Paek & Murray, 2021	1	1	1	2	1	1	1	UTD	0	1	N/A	1	1	UTD	UTD	0	68.75%
Paek, 2021	1	1	1	2	1	1	0	UTD	0	1	N/A	1	1	1	1	0	75.00%
Pakhomov et al., 2016 (Study 1)	1	1	1	1	1	1	1	UTD	0	1	N/A	1	1	1	1	1	81.25%
Pakhomov et al., 2016 (Study 2)	1	1	1	1	1	1	1	UTD	0	1	1	1	1	1	1	1	82.35%
Rofes et al., 2019	1	1	1	2	1	1	1	UTD	0	1	N/A	1	1	UTD	UTD	N/A	73.33%
Rofes et al., 2020	1	1	1	2	1	1	1	UTD	0	1	N/A	1	1	N/A	N/A	1	85.71%
Sailor et al., 2004 (Study 1)	1	0	1	2	1	1	0	UTD	0	1	N/A	1	1	0	UTD	0	56.25%
Sailor et al., 2004 (Study 2)	1	0	1	2	1	1	0	UTD	0	1	N/A	1	1	1	1	0	68.75%
Sailor et al., 2011	1	1	1	2	1	1	0	UTD	0	1	N/A	1	1	1	1	0	75.00%
Tiedt et al., 2022	1	1	1	2	1	1	1	UTD	0	1	N/A	1	1	1	UTD	0	75.00%
van den Berg et al., 2024	1	1	1	2	1	1	0	UTD	0	1	N/A	1	1	0	UTD	1	68.75%
Venneri et al., 2008	0	1	1	2	1	1	0	UTD	0	1	N/A	1	1	UTD	UTD	0	56.25%
Venneri et al., 2011	1	1	1	2	1	1	1	UTD	0	1	N/A	1	1	UTD	UTD	0	68.75%
Vita et al., 2014	1	1	1	2	1	1	1	UTD	0	1	1	1	1	UTD	UTD	1	76.47%
Vonk et al., 2023	1	1	1	2	1	1	1	1	0	1	1	1	1	N/A	N/A	1	93.33%
Wagner et al., 2020	1	1	1	2	1	1	1	UTD	0	1	N/A	1	1	1	1	1	87.50%
Wakefield et al., 2018	1	1	1	2	1	1	1	UTD	0	1	N/A	1	1	0	UTD	1	75.00%
Won et al., 2021	1	1	1	2	0	1	0	UTD	0	1	N/A	0	1	1	1	0	62.50%
Zabberoni et al., 2017	1	1	1	2	1	1	1	UTD	0	1	N/A	1	1	1	1	0	81.25%
*Studies that focussed on normal ageing*																	
Castro et al., 2021	1	1	N/A	2	1	0	1	N/A	N/A	1	N/A	1	1	1	1	0	84.62%
De Marco et al., 2021	1	1	N/A	2	1	0	1	N/A	N/A	1	N/A	1	1	1	1	1	92.31%
Hough, 2007	1	0	N/A	2	1	0	0	N/A	N/A	0	N/A	1	1	UTD	UTD	0	46.15%
Kavé et al., 2009 (Study 5)	1	1	N/A	2	1	1	0	N/A	N/A	1	N/A	1	1	UTD	UTD	0	69.23%
Murphy & Castel, 2021	1	1	N/A	0	1	1	1	N/A	N/A	1	N/A	1	1	1	1	0	76.92%
Taler et al., 2020	1	1	N/A	0	1	0	1	N/A	N/A	1	N/A	UTD	1	1	1	0	61.54%
Vita et al., 2014	1	1	N/A	2	1	1	1	N/A	N/A	1	N/A	1	1	UTD	UTD	1	84.62%
Vonk et al., 2019a, b	1	1	N/A	2	1	1	1	N/A	N/A	1	N/A	1	1	N/A	1	1	100%

Questions from the Downs and Black (1998) checklist were selected only if relevant to observational/quasi-experimental designs. Questions 4, 8, 9, 13, 14, 15, 19, 23, 24, 26, and 27 were discarded as they focus on aspects related to interventions. Study quality was exclusively evaluated in relation to the aspects of the articles that were of interest in this review (i.e. not necessarily in relation to the entire study), and in relation to the outcome variables described in Tables 2 and 3. UTD: “unable to determine” (i.e. it was counted 0 in the evaluation of study quality); N/A: “not applicable” (i.e. it was not counted in the evaluation of study quality). Quality levels were as follows: excellent ≥ 75%, moderate 50–74%, low 25–49%, and poor ≤ 25%

### Qualitative Synthesis – Neurodegenerative Conditions

**Fig. 2:**
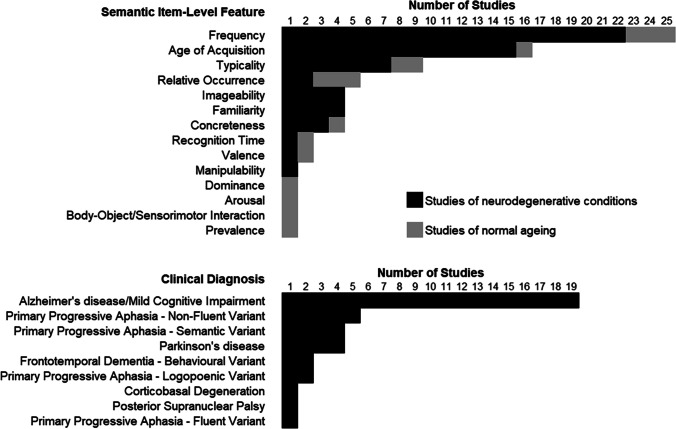
Count of studies that have investigated each item-level semantic feature and each clinical diagnosis

Twenty-seven studies/sub-studies either compared item-level CFT performance of individuals with a neurodegenerative condition to that of healthy adults or characterised the CFT performance of individuals with a neurodegenerative condition without the enrolment of a control group. These studies are summarised in Table 2. Among the various semantic item-level features, frequency was that most often scored, and the clinical continuum between MCI and AD was the diagnostic area most often investigated (Fig. 2). To facilitate consultation, the below sections are organised as a function of these two trends. Unless indicated, item-level scores were averaged across the entire CFT word list. While the findings associated with non-semantic features are also reported in the following sections, relational features such as those related to clustering and switching and features extracted from the Letter Fluency Test are only reported when these were combined with the features of interest as part of a composite variable or as part of a single inferential model.

**Table 2:** Qualitative synthesis of studies included in the review that focussed on neurodegenerative conditions

Study	Participants	AD Diagnostic Criteria	Country (test language)	Categories and modality	Features	Feature scoring	Inferential model	Findings
Beber et al., 2015***	• 19 mild AD • 16 moderate AD • 35 controls	McKhann et al., 1984 and DSM-IV criteria	Brazil (Portuguese -native)	Things that people do (1 min)—oral	• Frequency	Average of whole performance	One-way *ANCOVA* [age, education] and *post-hoc* Bonferroni-corrected* t*-tests	No significant effect of group was found
Binetti et al., 1995***	• 40 mild AD • 30 moderate-severe AD • 35 controls	McKhann et al., 1984	Italy (Italian)	Animals (1 min)—oral	• Frequency	Average of whole performance	One-way *ANOVA*; *post-hoc t*-tests	Both groups of individuals with AD named exemplars of a significantly higher frequency
Ferrante et al., 2024***	• 32 mild AD • 32 bvFTD • 27 controls	Dubois et al., 2007 McKhann et al., 2011	Latin America (Spanish)	Animals and letter P (1 min)—oral	• Frequency • Familiarity • Imageability • One-phoneme phonological neighbourhood • Phonemic length • Nodal granularity	Average of whole performance	Two-by-two (task-by-group) mixed *ANCOVA*s [sex, age, education] and *post-hoc* Tukey HSD tests. AD and bvFTD patients were analysed separately	*AD patients vs. controls*: a significant effect of group was found for frequency and granularity, with patients obtaining higher and lower scores, respectively. No group-by-task interaction effect was found. A significant interaction was found for phonological neighbourhood, with higher values recorded in the group of AD patients (than in controls) on the Animal Fluency task, and higher values recorded in the group of AD patients on the animal sub-task than the P sub-task. No other effects were reported *bvFTD patients vs. controls*: no effects emerged from the analyses
Forbes-McKay et al., 2005***	• 34 minimal AD • 39 mild AD • 23 moderate AD • 40 controls	McKhann et al., 1984	UK (English—native)	Animals and fruits (1 min)—oral	• Age of acquisition • Typicality • Frequency • Graphemic length	Average of whole performance; Average of the first 5 words per category	*MANCOVA* [age and education] and *post-hoc* Tukey tests	*Whole performance*: all AD sub-groups generated words that were significantly shorter, more typical, earlier acquired and more frequently used than those of the group of controls. Controls could be distinguished from each AD sub-groups in relation to age of acquisition, typicality and frequency. Controls could only be distinguished from mild and moderate (but not minimal) AD in relation to word length *First 10 words generated*: AD individuals generated words that were significantly more typical, earlier acquired and more frequently used (but not shorter) than those of the group of older controls
Henderson et al., 2023***	• 18 mild AD • 16 bvFTD • 26 svPPA • 26 nfPPA • 17 CBD • 36 PSP • 33 controls	McKhann et al., 2011	UK (English)	Animals and letter P (1 min)—oral	• Frequency *(PC 3)* • Imageability* (PC 2)* • Age of acquisition *(PC 2/3)* • Concreteness* (PC 2)* • Familiarity *(PC 3)* • Semantic diversity *(PC 2/3)* • Density of semantic neighbourhood *(PC 3)* • Graphemic length *(PC 1)* • Mean orthographic Levenshtein distance with 20 closest neighbours* (PC 1)* • Mean phonological Levenshtein distance with 20 closest neighbours* (PC 1)*	Average of whole performance across both fluency tasks; a principal component (PC) analysis was then run, and 3 components were extracted	One-way *ANOVA* and *post-hoc* Tukey’s HSD tests	Principal component (*PC*) 1 (lexical, non-semantic): a significant effect of diagnostic group was led by svPPA individuals who named shorter and less lexically complex words than PSP and CBD individuals; *PC 2* (semantic): a significant effect of diagnostic group was led by svPPA individuals who named less semantically rich words than AD and nfPPA individuals; *PC 3* (semantic) showed no group differences
Herrera et al., 2012	• 20 PD and no dementia • 20 controls	N/A	Spain (Spanish)	Animals, actions and supermarket words (1 min)—oral	• Frequency	Average of each category; patients were tested twice: ON and OFF dopaminergic medication	One-way *ANOVA*s and *post-hoc t*-tests	A significant effect was found for actions words’ frequency: patients OFF medication generated significantly more frequent words than controls
Hough & Givens, 2004	• 10 mild AD • 10 moderate AD • 10 controls	McKhann et al., 1984	USA (English)	Four “common” and four “goal-directed” categories (no time limits)—oral	• Typicality	Average of whole performance within each category type; proportion of words within “typicality bands” for each category type	Whole performance: two-way, group-by-category type *ANOVA*; typicality bands: three-way group-by-category type-by-typicality band *ANOVA* to investigate proportional typicality-based distribution of entries. *Post-hoc* Tukey HSD tests	Significant effects of “group” and of the “group-by-category type” interaction term were found. Differences were found among all groups, with controls generating less typical words than AD individuals, and mild AD individuals generating less typical words than moderate AD individuals; while words were more typical for the goal-directed categories than the common categories in the group of controls, no difference was found in the two clinical groups; a significant group by typicality band interaction was also found: moderate AD individuals generated a smaller proportion of band 4 and fewer band 1–2–3- exemplars
Jiskoot et al., 2023	118 first-degree relatives of mutation-carrying FTD patients followed up in time, i.e • 55 non-carrier controls • 63 mutation-carriers (i.e. 20 MAPT and 43 GRN) individuals, asymptomatic at study entry. Ten of these (i.e. 6 MAPT and 4 GRN) showed symptoms at follow ups (i.e. “phenoconverters”: 8 bvFTD and 2 nonfluent PPA)	N/A	The Netherlands (Dutch)	Animals (1 min)—oral	• Frequency • Age of acquisition	Average of whole performance	One-way *ANOVA*s (and Bonferroni-corrected *post-hoc* tests) at 5 timepoints	When phenoconverters were analysed as a single group, they generated words of higher frequency than controls at all timepoints (i.e. starting from 4 years before phenoconversion), and words acquired earlier in life only at phenoconversion and subsequent timepoints. No effect was found in mutation-carrying non-phenoconverters When MAPT and GRN phenoconverters were analysed separately, only MAPT phenoconverters showed significant changes, with words of significantly higher frequency and lower age of acquisition recorded at all timepoints. No effects were found for GRN phenoconverters, at any timepoints
Marczinski & Kertesz, 2006***	• 20 mild AD • 8 svPPA • 4 fluent PPA • 8 nfPPA • 20 controls	McKhann et al., 1984	Canada (English)	Animals and grocery items (1 min)—oral	• Frequency	Average of whole performance within each category type. Individuals with fluent PPA and nfPPA were combined in a single "PPA" group	One-way *ANOVA* and *post-hoc* comparisons; one-way *ANCOVA* [age, education, MMSE]	An effect of “group” was found for both categories. When *post-hoc* analyses were run: animals: differences in frequency were found across all groups aside from the PPA-AD comparison (i.e. controls < AD/PPA < svPPA); groceries: differences in frequency were found between the AD group and the other groups (AD > controls/svPPA/PPA)
Moreno-Martínez & Montoro, 2010 (cross-sectional findings only)	• 9 mild AD • 9 controls	McKhann et al., 1984 and DSM-IV criteria	Spain (Spanish)	14 categories and two domains: 7 living and 7 nonliving (1 min)—oral	• Age of acquisition • Familiarity • Manipulability • Typicality • Frequency • Graphemic length	Average of whole performance across all categories and within each domain	Hierarchical regressions to predict quantitative category fluency CFT performance within each group using item-level scores (block 1) and domain (block 2)	Age of acquisition, familiarity, and manipulability (and domain, from block 2) were significant predictors in both baseline models. Graphemic length was a significant predictor in AD individuals only. Frequency was not a significant predictor
Paek & Murray, 2021***	• 11 mild AD • 12 controls	McKhann et al., 2011	USA (English)	Things that people do (30 s)—oral	• Frequency, • Test-based age of acquisition, • Ratings-based age of acquisition, • Orthographic neighbourhood density • Phonological neighbourhood density, • Phonemic length • Syllabic length	Average of whole performance	Independent-sample *t*-tests	Frequency was significantly higher in the AD group. Rating-based age of acquisition was significantly higher in the group of controls. Phonemic and syllabic length were significantly higher in the group of controls. No differences were found in test-based age of acquisition, nor in phonological/orthographic neighbourhood density
Paek, 2021	• 15 mild amnestic AD • 17 controls	McKhann et al., 2011	USA (English—native)	Things that people do (30 s)—oral	• Valence	Average of whole performance	Independent-sample* t*-tests	Valence was significantly higher in the AD group than in the group of controls
Pakhomov et al., 2016 (study 1)***	• 50 AD • 71 MCI • 46 controls	DSM-IV criteria	USA (English)	Animals (1 min)—oral	• Frequency	Logarithm of the average of whole performance	One-way *ANOVA* and *post-hoc* Tukey HSD tests	AD individuals generated words of significantly higher frequency than MCI individuals and controls
Pakhomov et al., 2016 (study 2)	• 43 AD • 200 MCI • 213 controls	DSM-IV criteria	USA (English)	Animals (1 min)—oral	• Frequency	Logarithm of the average of whole performance	Mixed modelling of frequency as a function of the interaction between diagnostic category and time (i.e. slope of controls or the difference between the MCI/AD slope and that of controls), age, sex, years of education, density of perseverations and baseline/time-updated quantitative fluency scores	All variables were significant predictors in the model. Differences existed in longitudinal trajectories of frequency across groups, with AD individuals showing a significant upward trend, and MCI and control individuals showing minimal upward-directed changes
Rofes et al., 2019	• 10 lvPPA • 11 nfvPPA • 8 svPPA • 10 controls	N/A	USA (English—native)	Category Fluency-only model: animals, fruits and vegetables (1 min); combined Category-Letter Fluency model: also letters F, A and S (1 min)—oral	• Age of acquisition • Concreteness • Familiarity • Frequency • Imageability • Phonemic length • Semantic association • One-grapheme orthographic similarity • One-phoneme phonological similarity • Six error types (repetitions, fragments, phonological paraphasias, neologisms, wrong category, wrong letter)	Average of whole performance	Modelling of quantitative fluency scores (i.e. combined Category-Letter Fluency and Category Fluency only): Random-Forest-based ranking of significant features and confirmatory calculation of sensitivity and specificity scores, Conditional Inference Tree modelling and an *ANOVA* with *post-hoc* Tukey HSD tests	Combined Category-Letter Fluency model: group [sensitivity, specificity]: svPPA [0.44, 0.86]; lvPPA [0.34, 0.77]; nfvPPA [0.34, 0.74]; controls [0.83, 1]. Significant classifiers: quantitative scores, familiarity, phonemic length, frequency, age of acquisition, repetition count, concreteness, semantic association and imageability. Conditional Inference Tree model: beyond quantitative scores (which separated PPA individuals from controls, i.e. more/less than 75 words), familiarity was significantly higher in svPPA than in the other PPA groups (confirmed by ANOVA and *post-hoc* tests). Category Fluency-only model: Group [sensitivity, specificity]: svPPA [0.14, 0.78]; lvPPA [0.33, 0.75]; nfvPPA [0.31, 0.73]; controls [0.77, 1]. Quantitative scores, phonemic length, age of acquisition, semantic association, repetitions count, phonological paraphasias count. Conditional Inference Tree model: beyond quantitative scores (which separated PPA individuals from controls, i.e. more/less than 25 words), no predictor improved classification any further
Rofes et al., 2020	• 58 mild-to-moderate AD	McKhann et al., 1984	USA (English—native)	Animals (1 min)—oral	• Age of acquisition • Concreteness • Familiarity • Frequency • Imageability • Phonemic length • Semantic association • One-grapheme orthographic similarity • One-phoneme phonological similarity	Average of whole performance; features were computed in combination with cluster-based and switching-based features	Modelling of quantitative CFT scores: Random-Forest-based ranking of significant features and confirmatory Conditional Inference Tree modelling and Wilcoxon *post-hoc* tests to compare individuals who scored below vs. above	The order of importance of predictors of quantitative scores was: switches count, age of acquisition, frequency, familiarity, orthographic one-letter similarity, phonological one-letter similarity, phonemic length and mean cluster size. Conditional Inference Tree model: an interaction was found between number of switches and age of acquisition. *Post-hoc* comparisons: individuals scoring below the normative quantitative threshold made significantly fewer switches and generated words of earlier age of acquisition than individuals scoring within normality
Sailor et al., 2004 (study 1)	New York sub-cohort • 74 mild AD • 52 moderate AD • 78 controls Oregon sub-cohort • 32 AD • 37 controls	McKhann et al., 1984 and DSM-IV criteria	USA (English)	New York subcohort: male first names and footwear; Oregon subcohort: animals (1 min)—oral	• Relative occurrence	Average of the whole performance and average of the first three words	One-way *ANOVA*s	On average, individuals with AD (of either severity) generated more frequently occurring exemplars of footwear and animals than their respective controls (i.e. there was no effect on the male first names category). This was reflected by between-group differences in the average score across the entire performance. No differences were found when the analysis was limited to the first three words
Sailor et al., 2004 (study 2)	• 39 mild AD • 53 controls	McKhann et al., 1984 and DSM-IV criteria	USA (English)	Animals, fruits and vegetables (1 min)—oral	• Relative occurrence	Average of the whole performance, and cumulative probability of initial responses (i.e. the first words generated across the cohort)	One-way *ANOVA*s and a sign test for cumulative probability	Significant group differences were found when the average of the whole performance was analysed (within each category and across all categories combined). The probability was higher in the AD group for 29 of 62 initial responses. The cumulative probability for 25 of these 29 items was lower in the AD group (i.e. the probability was significantly different from chance level in the fruits and in the “vegetables” categories, but not in the “animals” category)
Sailor et al., 2011***	• 22 mild AD • 34 controls	McKhann et al., 1984 and DSM-III criteria	USA (English)	Animals, fruits and vegetables (1 min) and letters F, A and S—oral	• Age of acquisition • Frequency	Age of acquisition: average of the whole performance; frequency: average of the sum of log frequency and log-difference between the participant’s age and the age of acquisition of each word	Two-way mixed *ANOVA*, i.e. task type (Category Fluency and Letter Fluency) and diagnostic group). Age of acquisition was also analysed by calculating the residuals after frequency was regressed out (and the opposite was done in the analysis of frequency scores)	Age of acquisition was lower in the AD group. A significant task type-by-diagnostic group was found: Age of acquisition was lower for semantic categories and this difference was more pronounced in the AD group. After regressing out frequency, the effect of diagnosis was no longer significant, while the effect of the interaction was retained. Frequency scores were higher in the AD group, but no task type-by-diagnostic group was found. These findings did not change after controlling for age of acquisition
Tiedt et al., 2022	• 26 PD • 26 controls	N/A	Germany (German)	Vegetables (2 min) and animals/furniture (alternating—2 min), plus letter S (2 min) and letters G/R (alternating—2 min)—oral	• Frequency	Average of the whole performance and the difference between median of the first half and median of second half (i.e. frequency change)	Fluency type (Category-Letter)-by-alternation (yes–no)-by-diagnostic group mixed *ANOVA*. These analyses were run twice, with PD participants on and off medication. Fluency type (Category-Letter)-by-alternation (yes–no)-by-medication within-subject *ANOVA* to factor medication in, in the sole group of PD participants	No effect of diagnostic group (or of an interaction involving diagnostic group) emerged from the “medication-OFF” mixed *ANOVA*. An effect of group was found in the “medication-ON” *ANOVA*, with patients showing a smaller frequency change than control. A three-way interaction was also found in this model: in the analysis of Category Fluency tests only, a significant effect of diagnostic group was found (and no interaction); in the analysis of Letter Fluency a significant effect of the group-by-alternation was found (i.e. details not relevant to this review)
van den Berg et al., 2024	• 51 bvFTD • 27 svPPA • 25 nfPPA • 34 lvPPA • 25 controls	N/A	Netherlands (Dutch)	Animals (1 min)—oral	• Age of acquisition • Frequency • Graphemic length • One-phoneme orthographic similarity	Average of the whole performance	One-way *ANCOVA* [age, sex and number of CFT words] followed by *post-hoc* tests corrected for multiple comparisons. Linear regression [age and sex] to test the association between item-level features and cognitive composites	An effect of diagnostic group was found for frequency and age of acquisition. All clinical groups generated words that, on average, were of lower age of acquisition than controls, and this effect was significantly more pronounced in svPPA individuals than in the other clinical groups. Moreover, all clinical groups generated words that, on average, were more frequent than those generated by svPPA and control participants
Venneri et al., 2008***	• 25 mild AD • 25 controls	McKhann et al., 1984	UK (English)	Animals and fruits (1 min)—oral	• Age of acquisition • Typicality • Frequency • Graphemic length	Average of whole performance	One-way *ANOVA*	AD individuals generated words that, on average, were significantly more typical of their category and acquired significantly earlier in life
Venneri et al., 2011***	• 14 APOE-ε4 carriers amnestic MCI • 14 APOE-ε4 noncarriers amnestic MCI • 11 controls	N/A	Italy (Italian)	Animals and fruits (1 min)—oral	• Age of acquisition • Typicality • Graphemic length	Average of the whole performance	*ANCOVA* [education] and Scheffe *post-hoc* tests	A significant effect of group was found. The two groups of MCI individuals generated words that were of significantly lower age of acquisition than that of controls. No difference was found between the two MCI groups
Vita et al., 2014***	• 60 amnestic MCI • 20 mild-to-moderate AD • 25 young controls • 25 older controls	McKhann et al., 2011	Italy (Italian—native)	Birds and furniture (1 min)—oral	• Frequency • Typicality	Average of the whole performance. For the purpose of longitudinal analyses, the group of MCI individuals were split into a low-typicality and a high-typicality sub-groups	One-way *ANCOVA* [number of CFT words] and Sidak *post-hoc* tests. Chi square tests and logistic regression for the purpose of longitudinal analyses	A significant effect of group was found. AD individuals and MCI individuals generated words that, on average, were more typical than those generated by the two groups of healthy controls. The two clinical groups did not differ from one another. Fifteen aMCI individuals who converted to clinical AD after 24 months were part of the high-typicality group, and 5 were part of the low-typicality group. This difference was statistically significant. High-typicality was retained as significant predictor in the logistic regression modelling conversion
Vonk et al., 2023	• 583 individuals, cognitively healthy at baseline, and followed up in time	McKhann et al., 1984 and DSM-III criteria	USA (English or Spanish)	Animals (1 min)—oral	• Frequency • Age of acquisition • Recognition time	Average of the 10 “most difficult” words generated in relation to each feature for each feature (average of the 5 most difficult words and of all words as well)	Latent growth curve models inferring change in memory score, corrected for age and recruitment wave (models A), for all neurocognitive tests (models B) and quantitative score on the CFT (models C)	All baseline features were associated with memory decline (models A and B). Frequency and age of acquisition were associated with memory decline as per models C. Age was significantly associated with all features
Wagner et al., 2020	• 17 PD with left-sided symptoms • 17 PD with right-sided symptoms • 17 controls	N/A	USA (English)	Animals (1 min)—oral	• Frequency • Age of acquisition	Average of the whole performance	One-way *ANOVA* and Tukey *post-hoc* comparisons and one-way *ANCOVA* [other feature]	A significant effect of group was found on age of acquisition: PD individuals with right-sided symptoms generated words acquired earlier in life than controls. This effect persisted after controlling for frequency
Wakefield et al., 2018***	• 20 amnestic MCI • 20 functional memory disorder • 20 controls	N/A	UK (English)	Animals and fruits (1 min)—oral	• Age of acquisition	Average of the whole performance and average of first 5 words produced per category	*ANCOVA* [education] and Bonferroni *post-hoc* tests	A significant effect of group was found: control individuals and individuals with functional memory disorder generated words that were acquired significantly later in life. This was found when the average of the whole performance was analysed and when the first 5 words were analysed
Won et al., 2021***	• 17 MCI due to AD • 18 controls	Albert et al., 2011	USA (English)	Animals (1 min)—oral	• Frequency • Age of acquisition • Syllabic length	Average of the whole performance	Mixed group-by-timepoint *ANOVA* to test the effect of an exercise training programme on the features	No effect of group or of the group-by-timepoint interaction term
Zabberoni et al., 2017	• 20 PD • 18 controls	N/A	Italy (Italian)	Trees and furniture (1 min; version 1); colours and animals (1 min; version 2)—oral	• Typicality	Average of the first half and second half of the performance	Mixed group-by-treatment-by half performance *ANOVA*. PD individuals were tested ON and OFF medication and controls were similarly tested twice	No effect involving the variable group was significant

Correction factors are indicated in square brackets

*AD* Alzheimer’s disease, *CBD* cortico-basal degeneration, *GRN* granuline, *HSD* honestly significant difference, *lvPPA* primary progressive aphasia – logopoenic variant, *MAPT* microtubule-associated protein tau, *MCI* mild cognitive impairment, *MMSE* mini-mental state examination, *nfPPA* primary progressive aphasia – non-fluent variant, *PD* Parkinson’s disease, *PSP* progressive supranuclear palsy, *svPPA* primary progressive aphasia – semantic variant

^***^ identifies studies included in the two meta-analyses

#### Studies Carried Out in AD and MCI That Included Frequency Scores

**Fig. 3:**
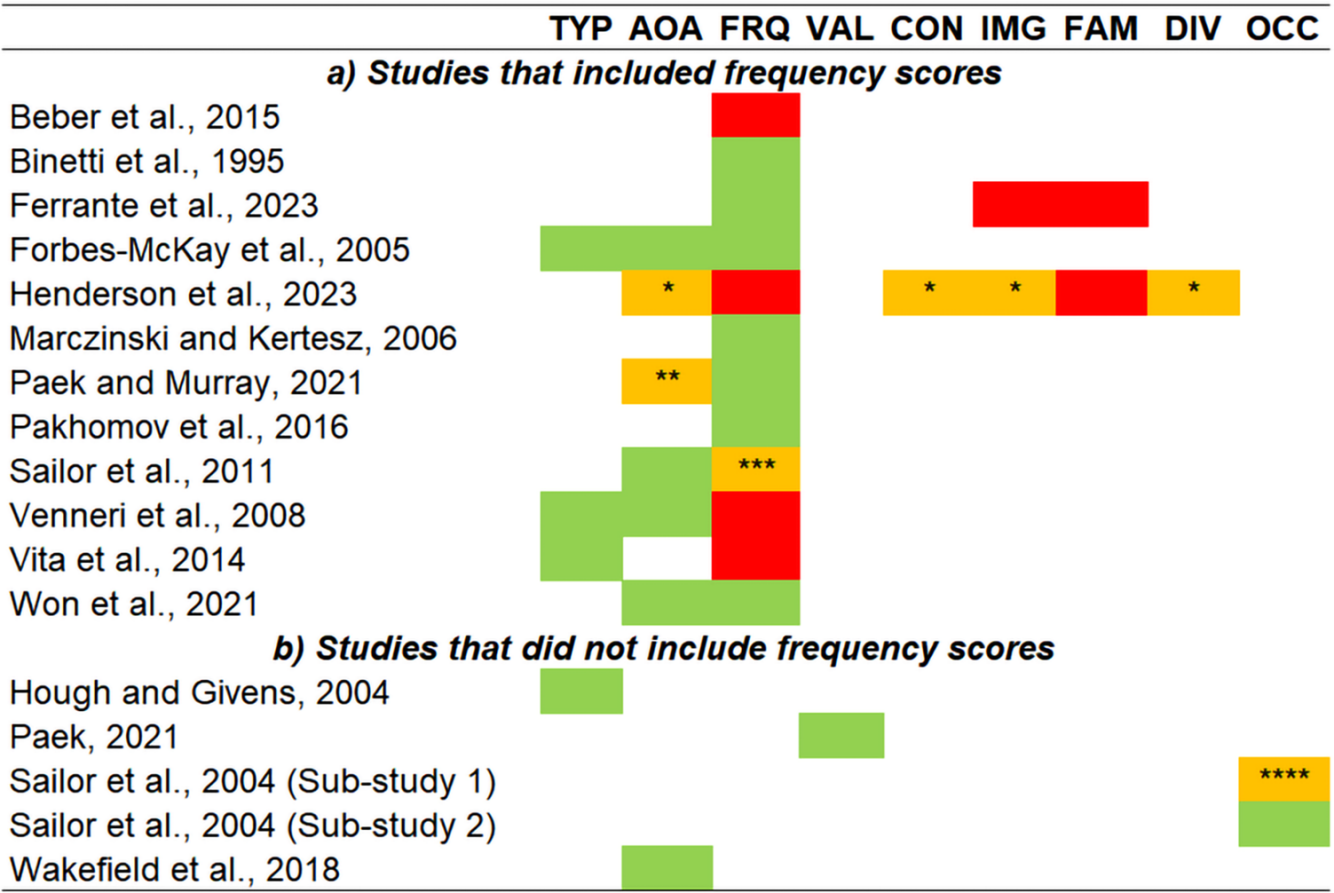
Effect of a clinical MCI-AD diagnosis on average item-level CFT words’ features. Studies based on MCI/AD vs. controls between-group differences only are reported. While significant and non-significant effects are reported in green and red, respectively, yellow cells indicate “incomplete” significance, as follows: * the group difference emerges in relation to the principal component on which the feature loads; ** the group difference emerges in relation to rating-based scoring, not test-based scoring; *** the group difference emerges only when the feature is scored for the CFT and Letter Fluency Test combined; **** the group difference emerges when the feature is scored in relation to two of the three CFT categories (but not in relation to the third one). Abbreviations: AOA, age of acquisition; CON, concreteness; DIV, semantic diversity; FAM, familiarity; FRQ, frequency; IMG, imageability; OCC, relative occurrence; TYP, typicality; VAL, valence

Twelve studies focussed on the clinical MCI-AD continuum, relying on a cross-sectional design. Binetti and colleagues (1995) reported that individuals with mild AD and individuals with moderate-to-severe AD generated words of higher *frequency* than controls (while the two clinical groups were not compared). Mini Mental State Examination scores, however, ranged between 30 and 22 in the group of controls, suggesting that no stringent clinical criteria had been applied in recruiting this group. A second study that uniquely focussed on frequency confirmed these results, reporting that individuals with AD (of no specific clinical severity) generated CFT words of higher frequency than those generated by controls and by individuals with MCI (Pakhomov et al., 2016). No significant difference between controls and MCI, however, was found. In a third study carried out in 5 distinct clinical groups (part of these findings is reported in the “Studies Carried Out in PD” section), Marczinski and Kertesz (2006) analysed CFT word frequency via a cross-diagnostic one-way *ANOVA* (analysing a group of mild-AD individuals, a group of controls, and three groups diagnosed with a PPA variant). They analysed each of their two CFT categories independently and found that, for both categories, individuals with mild AD generated words of higher frequency than controls. While the study by Forbes-McKay et al. (2005) investigated frequency of CFT words, they also scored *age of acquisition*, *typicality*, and, as a control non-semantic feature, *graphemic length*, in three groups of AD individuals (at minimal, mild, and moderate levels of severity) and a group of controls. Clinical patients generated words that were more frequent, more typical, acquired earlier in life, and shorter in their graphemic form. When, however, features were only scored (and averaged) in relation to the first 5 words generated per category, only the three semantic features (but not graphemic length) retained their significant difference (Forbes-McKay et al., 2005). The same four features were scored by Venneri and colleagues (2008) in two groups of mild-AD and control participants. They found that the mild-AD group generated words that were more typical and acquired earlier in life, but no difference in frequency was found. A lack of effect was also reported by Beber and co-workers (2015): frequency of CFT words was analysed in two clinical groups (of mild and moderate AD) and in a group of controls, but no effect of group was found in relation to words’ frequency. While the vast majority of the studies described in this section relied on categories such as “animals”, “fruits”, or “vegetables” (sometimes defined as “Noun Fluency”), the category investigated by Beber and colleagues (2015) was “things people do” (i.e. “Verb Fluency”). In a very recent study by Ferrante et al. (2024), the authors investigated words’ frequency, *imageability*, *familiarity*, phonemic length, phonological neighbourhood, and granularity in a group of people who received a biomarker-based diagnosis of AD. They documented significantly higher frequency and lower granularity in the words generated by the AD group and a task (i.e. Category vs. Letter Fluency)-by-group interaction indicating a larger phonological neighbourhood for CFT words among AD participants. An eighth study compared CFT performance of mild-AD individuals and controls by relying on Verb Fluency and analysing frequency, age of acquisition (measured in two distinct ways, i.e. retrospective “*rating-based*” scores and “*test-based*” indices derived from the active observation of children acquiring the word in “real life”), orthographic and phonological neighbourhood, and phonemic and syllabic length (Paek & Murray, 2021). Words generated in the clinical group were of higher frequency, earlier rating-based age of acquisition, and were longer in terms of phonemes and syllables. No difference was instead found between the two groups in the words’ test-based age of acquisition, nor in the two measures of lexical neighbourhood. Sailor and colleagues (2011) recruited two groups of mild-AD and control participants and administered both CFT and the Letter Fluency Test. Item-level scores calculated from the two fluency tests were analysed via a single inferential model. A test-by-diagnosis interaction was found in relation to age of acquisition: an earlier age of acquisition was recorded in relation to CFT words (compared with Letter Fluency words), and this difference was significantly larger in the clinical group. This effect was retained after regressing out frequency from each individual word. When frequency was analysed (this was scored out by summing up the log-transformed word’s frequency and the log-transformed difference between the word’s age of acquisition and the participant’s age), however, no effect of interaction was found. AD participants generated words that were of higher frequency, but this effect did not differ between the two fluency tasks, and these findings were retained after controlling for age of acquisition (Sailor et al., 2011). Vita and colleagues (2014) scored CFT frequency and typicality in two clinical groups (amnestic MCI and mild-to-moderate AD) and in two groups of controls (young and older). Words generated by the two clinical groups were of higher typicality than those generated by the two control groups (with no differences found between the two clinical groups, and no differences found between the two control groups). No effect, however, was found in relation to CFT words’ frequency. While the most common category used to test Noun Fluency is “animals”, participants in this study had been administered “furniture” and “birds” (i.e. a sub-category of “animals”). In an eleventh study, Won and co-workers (2021) tested the difference in frequency, age of acquisition, and syllabic length between a group of MCI individuals and a group of controls. Their design was based on a 3-month training programme consisting of walking sessions that was administered to both groups (i.e. no control condition was included) in order to model the group-by-timepoint interaction. Although no effect was reported in relation to timepoint or to the interaction term, an effect of the diagnostic group was visible for frequency and age of acquisition, with MCI individuals generating words that were more frequent and acquired earlier in life (Won et al., 2021. The authors did not report an effect of “group”, but group differences emerged from the calculation of the *t*-statistic based on means and standard deviations reported in relation to the baseline measurements). A recent study by Henderson and colleagues (2023), finally, combined the scoring of CFT and Letter Fluency by averaging item-level scores across both test performances. They scored words’ frequency, age of acquisition, imageability, familiarity, *concreteness*, *semantic diversity*, density of semantic neighbourhood, graphemic length, and both orthographic and phonological neighbourhoods in 7 clinical groups (i.e. part of these findings is reported in the “Studies Carried Out in PD” section). They then ran principal component analyses to identify three latent variables of interest accounting for semantic and non-semantic sources of variability. One of the two semantic components indicated that individuals with mild AD generated words that were semantically more complex than those of individuals with the semantic variant of PPA. No differences between AD individuals and controls emerged from these models, and no other effects involving the AD group were found in association with the other two components. A schematic colour-coded overview of the findings that emerged from these 12 publications is illustrated in Fig. 3a.

An additional two studies investigated frequency and other item-level features but did so via different inferential approaches. A study based on 18 participants (9 individuals with AD and 9 controls) and investigating 14 CFT categories (7 living, e.g. “flowers”; 7 non-living, e.g. “buildings”) focussed on frequency, age of acquisition, typicality, graphemic length, familiarity, and *manipulability*, not to study their average score but to predict quantitative CFT performance within each clinical group (Moreno-Martínez & Montoro, 2010). Age of acquisition, familiarity, and manipulability were significant predictors of CFT performance in both groups (with familiarity being the most important predictor), while graphemic length was a significant predictor of CFT performance in AD individuals only. Frequency was instead not a significant predictor. A further study was run with the purpose of predicting quantitative CFT performance: Rofes and colleagues (2020) analysed the CFT performance of a single group of participants diagnosed with mild-to-moderate AD, by scoring words’ age of acquisition, concreteness, familiarity, frequency, imageability, phonemic length, and orthographic and phonological neighbourhoods. In addition, each word was assigned to a sub-category (i.e. the category was “animals”, and 22 thematic sub-categories were defined) in order to score clustering and switching. The authors combined all these features in a Random Forest analysis to quantify their relative importance as predictor of CFT performance, and Conditional Inference Trees were applied to test for interaction effects. While number of switches and age of acquisition were the two best-performing predictors (the whole list is reported in Table 2), an interaction between the two was also reported: age of acquisition (i.e. above two split points of 4.64 and 4.14) predicted better CFT performance, but only for participants who showed 5.8 switches or more (Rofes et al., 2020). The numerical details reported by this study perfectly exemplify how unique each category is, with regard to clustering and switching.

Two further studies were carried out using a longitudinal design. A cohort of > 450 participants was recruited and followed up in time by Pakhomov and colleagues (2016) as part of the Mayo Clinic Study of Aging (the cross-sectional findings of this research are reported above, in this same section). A linear model was designed by these authors to analyse the trajectory of words’ frequency over time, and a mixed-effect term was added to test the interaction between timepoint and diagnostic status (i.e. healthy control, MCI, or AD). This interaction term emerged as a significant predictor, with findings revealing a significant effect of timepoint in the group of healthy controls (i.e. with CFT frequency significantly increasing from the baseline over the course of the four follow-up re-assessments) and a significant effect of the difference between the trajectory of controls and those of each group of patients, both considerably less steep (Pakhomov et al., 2016). Finally, a very recent study by Vonk et al. (2023) followed up a cohort of 583 individuals, healthy at baseline, over the course of 11 years, to model episodic memory decline (operationalised via change scores derived from performance on the Buschke Selective Reminding Test) via latent-growth curve models. They scored CFT words’ frequency, age of acquisition, and *recognition time* (see Box 2) at baseline (this last measurement was obtained from a large normative database), and each feature consisted of the average of the 10 most difficult words generated during the test. All baseline item-level features were significant predictors of memory decline, and this finding was confirmed even after controlling for all non-CFT neuropsychological test scores. When quantitative CFT scores were additionally added as correction factors, however, only frequency retained its significance (Vonk et al., 2023).

#### Studies Carried Out in AD and MCI That Did Not Include Frequency Scores

The findings reported in this section are illustrated in Fig. 3b. Hough and Givens (2004) investigated exclusively words’ typicality and did so by testing controls and individuals with mild and moderate AD (each of the three groups having a “*n* = 10” size) via a modified CFT consisting of 8 (i.e. 4 “regular” and 4 “goal-directed”) categories, with no time constraints. Goal-directed categories are “instrumental to achieving goals”, e.g. “things to take on a picnic” and are typically less consolidated within the semantic system than regular categories such as “sports” or “birds” (Hough & Givens, 2004). A significant effect of group was found, with words being significantly more typical in the mild-AD group and in the moderate-AD group. A group-by-category type interaction was also found, indicating that CFT words were more typical when the category was “goal-directed”, but this effect was only seen in the group of controls. These authors also assigned each word to one of seven category-specific “typicality bands”, with the purpose of characterising the effect of disease on this distribution. A significant group-by-typicality band interaction was found, indicating that individuals with moderate AD generated significantly fewer words belonging to the three more typical bands and significantly more words within the fourth, “mid-range” band (Hough & Givens, 2004). This is the only publication indicating that individuals with a neurodegenerative disease generate more untypical words than healthy controls. Words’ *valence* was investigated by Paek (2021), who administered a 30-s version of the CFT to individuals with mild AD and controls. The statistical comparison indicated that AD individuals generated significantly fewer “things people do” (Verb Fluency), but these were characterised by a higher emotional valence. The manuscript by Sailor and colleagues (2004) reports the findings of two distinct sub-studies of CFT words’ relative occurrence (labelled “typicality” by the authors). In their first sub-study, they analysed two separate cohorts to characterise the difference between AD individuals and controls. All groups of AD individuals (of varying clinical severity) generated words of higher relative occurrence. This, however, was only reported in association with two of the three categories (i.e. “footwear” and “animals”) but not in relation to “male first names”. In a parallel set of analyses, the authors also limited their scoring to the first 3 words generated during CFT performance, but none of the resulting effects was significant. In their second sub-study, they focussed on the cumulative probability of generating 29 individual words that were more common as initial responses in the AD group. An effect of diagnostic group on these words’ relative occurrence was confirmed for all three target categories (“animals”, “fruits”, and “vegetables”), and, in addition, the cumulative probability of AD-related initial responses was significantly lower in the AD group for 25 of the 29 words (Sailor et al., 2004). In a study carried out in three diagnostic groups (amnestic MCI, functional memory disorder, and controls), Wakefield et al. (2018) tested the between-diagnosis difference in words’ age of acquisition. Individuals diagnosed with amnestic MCI named words acquired significantly earlier in life than the other two groups (who did not show any difference between each other). This statistical effect was confirmed when age of acquisition was averaged in relation to the first five CFT entries only. A final study carried out exclusively in a cohort of MCI participants (and, for this reason, not included in Fig. 3b) investigated the effect of the apolipoprotein ɛ4 allele (i.e. an established risk factor for late-onset AD) on age of acquisition, typicality, and graphemic length. Two groups of MCI participants (one of ɛ4 carriers, one of ɛ4 non-carriers) and a group of controls were recruited, and item-level analyses of CFT performance showed that both MCI groups generated words that are acquired earlier in life than those generated by controls, while no difference was documented between the two MCI groups, nor in typicality or graphemic length (Venneri et al., 2011). It is particularly interesting to acknowledge that ɛ4 non-carriers showed a non-significant trend towards less typical words and words acquired later in life compared to ɛ4 carriers, in spite of their considerably shorter (4.72 years less, on average) educational attainment.

In summary, 21 studies have characterised CFT performance adopting an item-level scoring approach to describe changes in semantic memory in MCI and AD. As shown in Fig. 3, the vast majority of these studies reported impoverished lexical-semantic output in these individuals in relation to a clinical trait of relevance in at least one of the features investigated.

#### Studies Carried Out in PD

Four studies were included in the qualitative synthesis in relation to this diagnosis, all carried out in samples of individuals with normal cognitive functioning. A first study recruited healthy controls and individuals with PD and allocated the latter to two groups based on symptom laterality (i.e. left-sided or right-sided). Frequency and age of acquisition of CFT words were analysed: PD individuals with right-sided symptoms generated words that were of an earlier age of acquisition than controls, and this effect was still significant after controlling for frequency (Wagner et al., 2020). The authors postulated a link between right-sided symptoms and the more pronounced involvement of the left cerebral hemisphere, known to support linguistic functioning. The other three studies tested PD participants twice, ON- and OFF medication. Zabberoni and colleagues (2017) administered the CFT to individuals with PD and controls (who were also tested twice) and scored words’ typicality by independently averaging the scores of the first and of the second half of performance (alternative CFT categories were used to allow repeated testing). An *ANOVA* was run to model item-level features as a function of “group”, “treatment”, and “performance half”, but none of the effects (including interaction effects) involving the variable “group” emerged as significant (Zabberoni et al., 2017). In the study by Herrera and colleagues (2012), the group of controls completed the CFT only once, and no alternative CFT categories were used in the two PD conditions. Frequency was scored in relation to three categories (i.e. “animals”, “supermarket items”, and “things you can do”), which were analysed via separate models. The findings indicate an effect of diagnosis, but only for “things you can do” (Verb Fluency), with frequency scores being significantly higher in PD individuals OFF medication than in controls (Herrera et al., 2012). The authors of this study addressed the potential impact of pseudoreplication (as the ON and OFF conditions, despite not being independent of one another, were analysed as part of an independent-sample *ANOVA*) by confirming the absence of an effect of task repetition via dedicated *a priori* analyses in which each fluency measure was modelled as a function of the order of conditions, i.e. first ON vs. first OFF. Finally, the study by Tiedt and co-workers (2022) investigated the frequency of words generated by PD individuals and controls during two versions of the CFT and of the Letter Fluency Test: a “classic” single-category/letter version and a “switching” version consisting of alternating words belonging to one of two categories/starting with one of two letters. Two aspects of frequency were scored: the global average and the difference between the median of the first half and the median of the second half (i.e. “frequency change”). Three sets of inferential models were run: fluency type-by-version-by-group *ANOVA*s (ON medication and, separately, OFF medication) and, within the group of PD individuals, fluency type-by-version-by-medication status *ANOVA*s. The findings indicated smaller frequency change scores in patients ON medication than control. Moreover, a three-way interaction was also found in this analysis. This was followed up by *post-hoc ANOVA*s, which revealed an effect of group in relation to CFT frequency measures (Tiedt et al., 2022).

In conclusion, these four studies provide significant yet modest evidence of a decline of semantic processing in PD when assessed via item-level scoring of CFT performance, with a modulatory role played by adherence to medication and by other clinical and methodological aspects such as symptom laterality, CFT performance half, and the use of specific categories.

#### Studies Carried Out in Other Neurodegenerative Conditions

Six studies are reported in this section (the findings outlined in three of these are also partly reported in the “Studies Carried Out in AD and MCI That Included Frequency Scores” section). Marczinski and Kertesz (2006) recruited participants with a diagnosis of PPA (semantic PPA, fluent PPA, and non-fluent PPA; fluent and non-fluent PPA individuals were merged in a single group) and compared them with a group of controls, analysing word frequency within two categories (which were analysed independently). When the “animals” category was analysed, people with semantic PPA showed higher frequency scores than the other PPA group which, in turn, showed higher frequency scores than controls. When “grocery items” were instead analysed, no between-group differences were found, and the authors suggested this may have been due to higher levels of variability for frequency applied in relation to this category because of the use of strategies based on autobiographical memory or to a wider neurological mapping of this category’s representations, as grocery items intersect a wide range of other categories (Marczinski & Kertesz, 2006). Van den Berg et al. (2024) scored frequency, age of acquisition, graphemic length, and orthographic neighbourhood in a group of controls and in four groups of participants diagnosed with the behavioural variant of frontotemporal dementia (bvFTD), semantic PPA, non-fluent PPA, or logopoenic PPA. An effect of group was only found in relation to frequency and age of acquisition: each clinical group showed lower age of acquisition than controls, and, additionally, this effect was significantly more pronounced in the group with semantic PPA than in each of the other clinical groups. Frequency, on the other hand, was significantly higher in all clinical groups apart from those with semantic PPA, who scored instead at the same level of controls (Van den Berg et al., 2024). In a third study carried out in individuals diagnosed with these same four clinical profiles, Rofes et al. (2019) averaged item-level properties of the CFT and of the Letter Fluency Test combined (and, in parallel, of the CFT on its own) and applied machine-learning methods (i.e. a Random Forest analysis) to test diagnostic classifications. They scored words’ age of acquisition, concreteness, familiarity, frequency, imageability, phonemic length, and orthographic and phonological neighbourhood. In addition, they also included standard quantitative scores and assessed semantic associations of retrieved words and six types of errors made during the CFT. When features were calculated on both fluency tests combined, quantitative scores and familiarity were the top two classifiers (the whole list is reported in Table 2). Conditional Inference Trees then identified an interaction between these two predictors, with familiarity contributing to classification accuracy only for participants who named 75 words or less. As six fluency subtests were administered (3 letters and 3 categories), the combination of the two tests does not allow to understand which of the two contributed the most to the classificatory outcome. When classification was uniquely based on the CFT, quantitative scores and phonemic length were the best two classifiers (the whole list is reported in Table 2), but no interaction was identified (Rofes et al., 2019). Henderson et al. (2023) compared the performance of a group of controls and 5 clinical groups, with diagnoses of bvFTD, semantic PPA, non-fluent PPA, cortico-basal degeneration, and progressive supranuclear palsy. The authors calculated words’ frequency, age of acquisition, imageability, familiarity, concreteness, semantic diversity, density of semantic neighbourhood, graphemic length, and orthographic and phonological neighbourhood and ran principal component analyses to describe group difference along three latent components. The first lexical, non-semantic component showed an effect of group, with individuals with semantic PPA naming words that were lexically less complex than those named by individuals with cortico-basal degeneration or progressive supranuclear palsy. The second, semantic component showed a similar effect of group, and it was again individuals with the semantic form of PPA who showed reduced semantic complexity than individuals with the non-fluent form of PPA. No effect, finally, was found in relation to component number 3 (Henderson et al., 2023). In a fifth study, Ferrante and colleagues (2024) compared a group of individuals diagnosed with bvFTD with a group of controls: they analysed frequency, imageability, familiarity, phonological neighbourhood, phonemic length, and *granularity* of CFT and Letter Fluency words but found no significant effects in this diagnostic group. The sixth and final study is a cohort-based initiative that enrolled first-degree family members of individuals with a diagnosis of bvFTD/PPA and a mutation in the “Microtubule-Associated Protein Tau” (MAPT) or “Granuline” (GRN) gene (Jiskoot et al., 2023). These individuals, who were all healthy at study entry, were followed up at multiple timepoints in order to monitor symptom onset (i.e. “phenoconversion”). The average frequency of CFT words generated by phenoconverters was significantly higher than that of control mutation-noncarriers at all timepoints, starting at 4 years before symptom onset. Words’ age of acquisition did not differ between the two groups at the presymptomatic stages, but phenoconverters generated words that were, on average, acquired earlier in life, in relation to the onset of symptoms (and continued doing so at subsequent follow-ups). When MAPT and GRN mutation carriers were analysed separately, the former showed significant differences in words’ frequency and age of acquisition at all timepoints, while the latter did not show any differences. No effects, finally, were instead reported in mutation-carriers non-phenoconverters (Jiskoot et al., 2023). This study complements the research presented in the rest of the section, as diagnostic status at baseline was based on genetic, rather than clinical variability.

While the studies reported in this section are based on diagnostic variability, with limited evidence available for certain forms of neurodegeneration, the majority of findings point towards impoverished item-level CFT scores in these conditions, with a particularly harsh effect observed in the semantic form of PPA.

### Qualitative Synthesis – Normal Ageing

Eight studies/sub-studies (schematised in Table 3) investigated the effects of ageing on item-level scores in healthy adults, either via a comparison between a group of young adults and a group of older adults or via a correlational model run between item-level features and age. In the oldest of these studies, Hough (2007) recruited 3 groups of adults (young, middle-aged, and older) and scored typicality of CFT words generated in response to four “common” and four “goal-directed” categories (no time limit was given). No effects emerged from the two-by-three, category type-by-group *ANOVA*. Words were then assigned to one of six typicality bands to analyse whether the predictors influenced this distribution. A significant three-way (group-by-category type-by-typicality band) interaction was found, indicating that older adults generated a higher proportion of words belonging to the most typical band and fewer words distributing in the second and third most typical bands, and this effect was significantly more pronounced in relation to the “common” categories. Two years later, Kavé and colleagues (2009) published a study carried out in a cohort of 136 adults subdivided into six age groups. In one of their sub-studies, they scored the relative occurrence of words generated by the youngest and oldest groups, counting the number of single-occurrence entries. The oldest group generated significantly more single-occurrence words, and, across the entire cohort, the number of single-occurrence words was positively correlated to age. In their study described in the “Studies Carried Out in AD and MCI That Included Frequency Scores” section, Vita et al. (2014) compared words’ frequency and typicality of younger and older controls (“items of furniture” and “birds” were administered), reporting significant differences neither in the number of words nor in item-level features. Taler and colleagues (2020) studied the association between item-level (frequency and orthographic neighbourhood) and other (pairwise similarity and the number of semantic sub-categories) features, and age, and did so in two large cohorts of ~ 6,000 adults each (one of adults aged 60 or below, one of adults aged 61 or above). Age was positively correlated to frequency and pairwise similarity in both cohorts, and both *z*-converted correlation coefficients were significantly stronger in the older cohort. The study by Castro and colleagues (2021) investigated written fluency for 70 distinct categories in three different age-related groups (young, middle-aged, and older). They scored the relative occurrence of words to quantify, within each category, words’ “type-to-token ratio” and “idiosyncratic type-to-total ratio”, where “type” identifies an entry named by at least one participant (and “idiosyncratic type” an entry named solely by one participant), and “token” identifies the number of participants who named that word. Older adults showed a lower type-to-token ratio than the other two groups, while no difference in idiosyncratic type-to-total ratio was recorded. The study by Murphy and Castel (2021) analysed written, 5-min Category Fluency in two large (*n* ~ 100) groups of young and older adults. They scored the relative occurrence of words and identified those generated by 5% or less of the cohort (these were labeled “original” entries). In addition, they also scored the serial recall order of words, i.e. the serial position at which each word was retrieved during performance. No difference in the relative occurrence of words (or original words) was reported between the two groups (but a significant positive correlation between age and average relative occurrence, however, was found across the entire cohort). A significant association was found between serial recall order and relative occurrence (i.e. indicating the tendency to generate words that are increasingly difficult), but this was reported for the whole cohort and in the group of older adults only (Murphy & Castel, 2021). No confounding variables, however, were used in this study. The serial recall order was studied in more depth by De Marco et al. (2021), who scored item-level typicality, frequency, age of acquisition, concreteness, prevalence, recognition time, body-object interaction, valence, arousal, dominance, graphemic length, syllabic length, consonant-to-vowel ratio, phonological complexity, and two indices of the orthographic neighbourhood. They assessed CFT performance in two groups (one young and one older) of adults and calculated the correlation coefficient between serial recall order and each of the above features. Only one of these (*z*-converted) coefficients was significantly different between the two groups: that between serial recall order and valence. Young adults generated more pleasant words at the start of the performance and showed then a drop in valence during the rest of the performance, that was significantly steeper than that shown by older adults. These authors also studied the network properties of item-level features (and, specifically, of serial recall order) using graph theory. Serial recall order had a significantly higher “degree” and a significantly weaker “betweenness centrality” in the group of older adults, indicating more significant correlations with item-level features and a weaker relevance within the overall network, respectively, while no differences were recorded in local or global efficiency metrics (De Marco et al., 2021). In an eighth publication that concludes this section, Vonk and colleagues (2019b) focussed on the apolipoprotein ɛ4 allele and characterised frequency in CFT performance of a cohort of adults aged above 54 years by analysing word frequency. A non-significant correlation between frequency and age was reported in the cohort. When the ɛ4 allele was investigated, frequency (but not quantitative CFT performance) was a significant predictor of genetic status. Furthermore, a group-by-time interval emerged from growth-curve models aimed at characterising performance across the six consecutive 10-*s* intervals: while no difference in frequency was found for the first interval, ɛ4 carriers generated words of higher frequency within each of the other five intervals (Vonk et al., 2019b). Although APOE and age are distinct variables, these findings are of interest because APOE variability is one of the best-established variables that influence the trajectory of neurological ageing.

**Table 3:** Qualitative synthesis of studies included in the review that focussed on normal ageing

Study	Participants	Country (test language)	Categories and modality	Features	Feature Scoring	Inferential model	Findings
Castro et al., 2021	• 83 young adults • 79 middle-aged adults • 84 older adults	USA (English—native)	70 distinct categories (30 s each)—written (typed)	• Relative occurrence	Category-specific “type-to-token” ratio Category-specific “idiosyncratic type-to-total” ratio	3 × 70 Friedman’s test and *post-hoc* symmetry tests	The group of older adults had a lower type-to-token ratio than the groups of middle-aged adults and younger adults (no difference was found between middle-aged and younger adults) No age-dependant difference was found in the idiosyncratic type-to-total ratio
De Marco et al., 2021	• 45 young adults (aged 18–21) • 45 older adults (aged 70 and above)	UK (English—native)	Animals and fruits (1 min)—oral	• Serial recall order • Typicality • Frequency • Age of acquisition • Concreteness • Prevalence • Recognition time • Body-object interaction • Valence • Arousal • Dominance • Graphemic length • Syllabic length • Consonant-to-vowel ratio • Phonological complexity • In-list Levenshtein distance • One-grapheme dictionary Levenshtein distance	Correlation between serial recall order and each feature and graph theory modelling	One-way *ANCOVA* [education, MMSE and number of CFT words] and “5-word interval” *post-hoc* *ANCOVA*s [education ad MMSE]	Young adults had a significantly weaker “serial recall order-valence” correlational score indicating a steeper trend towards increasingly less pleasant words in this group. Words 1–5 generated by young adults were significantly more pleasant than words 1–5 generated by older adults; serial recall order in older adults was characterised by a higher “degree” and smaller “betweenness centrality” than in younger adults. The remainder of the models was not significant
Hough, 2007	• 20 young adults • 20 middle-aged adults • 20 older adults	USA (English)	Four “common” and four “goal-directed” categories (no time limits)—oral	• Typicality	Average of the whole performance within each category type; proportion of words within “typicality bands” for each category type	Whole performance: two-way, group-by-category type *ANOVA*; typicality bands: three-way group-by-category type-by-typicality band *ANOVA*. *Post-hoc* Tukey HSD tests	No group-related effect emerged from the analyses of all words; group-by-typicality band and group-by-typicality band-by-category type interactions were found from the second analysis: in “common” categories, older adults generated a higher proportion of words of the most typical band and a lower proportion of words of the second and third most typical bands
Kavé et al., 2009 (Study 5)	136 adults subdivided into 6 age groups Results of study 5:—30 youngest adults—30 oldest adults	Israel (Hebrew)	Animals, fruits/vegetables and vehicles (1 min)—oral	• Relative occurrence	Number of single-occurrence words	Between-group *t*-test between the youngest and the oldest group of the cohort	Older adults generated more single-occurrence words. The number of single-occurrence words was positively associated with age across the entire cohort
Murphy & Castel, 2021	• 98 young adults • 96 older adults	USA (English)	Animals (5 min) – written (typed)	• Relative occurrence • Serial recall order	Average of the whole performance, and sum of “original” entries (i.e. generated by < 5% of the cohort)	Between-group *t-*test between the youngest and the oldest group of the cohort, and correlation between frequency and serial recall order	No differences in relative occurrence (i.e. average of performance or sum of original entries) were found between younger and older adults. A significant association was found between serial recall order and relative occurrence in the entire cohort and in the group of older adults only
Taler et al., 2020	Sub-study based on group comparisons: • 6764 cognitively normal adults aged 61 or above • 5922 adults aged 60 or below	Canada (English)	Animals (1 min)—oral (part over the phone)	• Frequency • One-grapheme orthographic similarity • Item-to-item pairwise similarity • Number of “Troyer categories” (i.e. semantic clusters)	Average of the whole performance	Correlational models (Pearson’s *r*) tested the associations between semantic features and age, within each age group. Between-group comparisons were then run to compare the two age groups after *r*-to-*z* transformations	Age was positively associated with average pairwise similarity and frequency in both age groups. Both coefficients of correlation were statistically stronger in the group of older adults
Vita et al., 2014	• 60 amnestic MCI • 20 mild-to-moderate AD • 25 young controls • 25 older controls	Italy (Italian—native)	Birds and furniture (1 min)—oral	• Frequency • Typicality	Average of the whole performance	One-way *ANOVA* and Sidak *post-hoc* tests	A significant effect of “group” was found. The two group of controls did not show any significant differences
Vonk et al., 2019a, b	• 81 APOE ɛ4 carriers • 145 APOE ɛ4 non-carriers	USA (English—native)	Animals (1 min)—oral	• Frequency	For the analysis of age-dependant effects: average of whole performance; for the analysis of ɛ4-dependant effects: average of whole performance and average of each 10-s interval	For the analysis of age-dependant effects: correlation between age and linguistic variables; for the analysis of ɛ4-dependant effects: logistic regression with ɛ4 status as outcome and quantitative and item-level CFT scores as predictors. Additionally, growth curve models [number of CFT words] were designed to test the effect of ɛ4 on quantitative and item-level scored throughout the six 10-s intervals	Age was not correlated with mean frequency. Quantitative performance was not a predictor of ɛ4 status but frequency was (with ɛ4 carriers tending to show higher frequencies than non-carriers). Growth curve models indicated no differences (nor changes) in the number of words across the six intervals. Conversely, an effect of the group-by-time interaction was reported for frequency: no difference in frequency was recorded in the first 10-s interval, while a difference emerged in the other five intervals (i.e. ɛ4 carriers generating words of higher frequency)

Correction factors are indicated in square brackets

*HSD* honestly significant difference, *MMSE* mini-mental state examination

In summary, although the inferential models run in these eight studies did highlight an effect of age in some item-level features of CFT performance, a large portion of the analyses revealed no association between these indices and age.

### Post-Hoc Meta-Analysis of Frequency and Age-of-Acquisition Ratings in AD and MCI

As shown in Fig. 2, frequency and age of acquisition were the features most commonly scored by clinical researchers. As these are two candidate features of simple operationalisation and with a potential application in the clinical setting, we decided to investigate them further with meta-analytical procedures, with a selective focus on the MCI-to-AD continuum. A total of 14 studies (12 investigating frequency and 8 investigating age of acquisition) investigating group differences between a clinical sample and a group of controls were considered for inclusion in two distinct meta-analyses. Methodological quality (Table 1), demographic factors calculated in the clinical group (i.e. age, education level, and performance on the Mini Mental Score Examination or other screening measure of cognitive severity), and CFT-related variables (number of categories tested and cross-diagnostic differences in quantitative scores) were identified as moderators of interest and extracted from each study, together with means and standard deviations of item-level features in each group. When studies assessed more than one clinical group (i.e. four studies in total), that at the mildest level of severity was selected to be included in the meta-analytical model. This choice was in line with the potential use of item-level CFT scores for early-stage disease detection. Moreover, individuals at more severe stages of AD dementia tend to generate a considerably smaller number of words, e.g. 3.5 (Binetti et al., 1995) or 4.28 (Beber et al., 2015), and, as a consequence, item-level averages may be less informative. Corresponding authors were contacted to request any missing information. The Supplementary Information section includes a description of data transformation processes applied to homogenise the variables across studies. Cross-diagnostic differences in quantitative scores were added to the models since previous meta-analyses demonstrated a strong effect of AD diagnosis on quantitative CFT scores (Henry et al., 2004; Laws et al., 2010). In both cases, this effect was interpreted as partly due to cross-diagnostic differences in executive processing. The function of this additional moderator was thus to regress out the portion of variability of quantitative scores associated with executive processing.

All meta-analytical procedures were run with ProMeta (version 3.0). Frequency and age-of-acquisition scores were defined as outcomes, and diagnostic status (i.e. MCI/AD dementia vs. normal controls) was selected as the predictor of interest. All aforementioned moderators were included in both analyses. Random-effect models were thus designed, and the effect direction was set as “positive” for frequency (as MCI/AD participants tend to generate words of higher frequency than controls) and “negative” for age of acquisition (as MCI/AD participants tend to generate words acquired earlier in life than controls), in order to test one-tailed hypotheses.

**Fig. 4:**
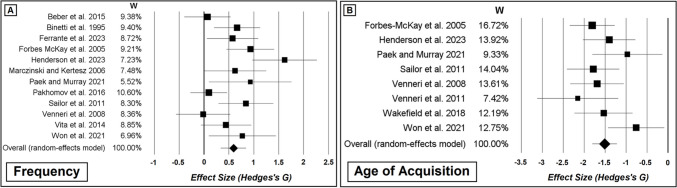
Forest plots summarising the effect of clinical diagnosis (i.e. AD/MCI vs. controls) on item-level scores. Effect sizes calculated from between-group comparisons of frequency scores are positive as MCI/AD participants tend to generate words of higher frequency than controls. Effect sizes calculated from between-group comparisons of age-of-acquisition score are negative, as MCI/AD participants tend to generate words of the earlier age of acquisition than controls. *W* indicates the proportional weight of each study

A total of 735 participants (385 with MCI/AD and 350 controls) were included in the analysis of frequency. The resulting effect size of the model (*Hedges’s G*) was equal to 0.59 (upper and lower limit: 0.34 and 0.85) and was significant at a *p* < 0.001 (Fig. 4a). Both the Egger’s linear regression test and the Begg and Mazumdar’s rank correlation test were non-significant (*p* = 0.051 and 0.055, respectively), indicating no publication bias. Significant heterogeneity was found across publications, with a *Q* value equal to 29.69 (*df* = 11, *p* = 0.002). *Tau* and *Tau-squared* coefficients (indicative of the standard deviation and variance of the true effect) were equal to 0.35 and 0.12, respectively, and the *I-squared* coefficient, indicative of the squared ratio between the precision interval of the effect and the dispersion of the effect across studies, was equal to 62.95. One study (Henderson et al., 2023) was identified as a potential outlier, with a standardised residual significant at a *p* = 0.008). The analyses were thus re-run without including data from this publication, but the resulting effect size (0.50) retained its significance at a *p* < 0.001. Removing this study, however, resulted in a considerable reduction of heterogeneity (*Q* = 18.19, *df* = 10, *p* = 0.052).

**Fig. 5:**
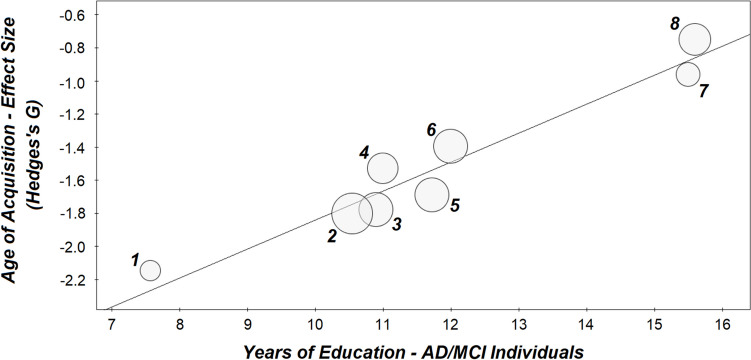
Linear association between the average educational attainment calculated in the group of participants with MCI/AD (i.e. moderator in the meta-analysis of age of acquisition values) and study effect size. Individual studies are numbered: (1) Venneri et al. (2011); (2) Forbes-McKay et al. (2005); (3) Sailor et al. (2011); (4) Wakefield et al. (2018); (5) Venneri et al. (2008); (6) Henderson et al. (2023); (7) Paek and Murray (2021); (8) Won et al. (2021)

A total of 354 participants (193 with MCI/AD and 161 controls) were included in the analysis of age of acquisition. *Hedges’s G* was equal to − 1.51 (upper and lower limit: − 1.80 and − 1.21) and was significant at a *p* < 0.001 (Fig. 4b). Both the Egger’s linear regression test and the Begg and Mazumdar’s rank correlation test were non-significant (*p* = 0.767 and 0.458, respectively), indicating no publication bias. No significant heterogeneity was found across publications, with a *Q* value equal to 10.52 (*df* = 7, *p* = 0.161). *Tau*, *Tau-squared*, and *I-squared* coefficients were equal to 0.24, 0.06, and 33.47, respectively. One study (Won et al., 2021) was identified as a potential outlier, with a standardised residual significance at a *p* = 0.017). As this was the study with the smaller effect size (i.e. the closest to non-significance), the analyses were not re-run without including data from this publication. Two moderators were reported as having a significant association with *Hedges’s G*: the number of categories tested, i.e. regression equation: *G* =  − 0.72 + (− 0.44 × number of categories), *p* = 0.036; and educational attainment of MCI/AD participants, i.e. regression equation: *G* =  − 3.60 + (0.18 × years of education), *p* < 0.001. The more categories tested, the larger the effect expressing a between-group difference in average age of acquisition of words. The more educated the group of MCI/AD participants, the smaller the effect expressing a between-group difference in average age of acquisition of words (Fig. 5).

As the results were characterised by a clear directionality (Fig. 4), with no significant effect recorded in the opposite direction (i.e. individuals with MCI/AD generating words of higher semantic complexity), this was interpreted as objective evidence of *certainty* for each of the two outcomes.

The number of studies investigating frequency and/or age of acquisition in other clinical groups (i.e. PD, bvFTD, svPPA, nfPPA, and lvPPA) was reviewed to consider further meta-analytical models. This number ranged from two to four, with overall sample sizes between *n* = 79 and *n* = 184 (i.e. corresponding to 10.7% and 25% of the sample included in the meta-analysis of frequency scores described above). As a result, no further analyses were run.

## Discussion

Item-level approaches have been studied for several decades in relation to CFT scoring to help characterise decline in SM in normal ageing and in individuals with suspected or clinically confirmed neurodegeneration. Although standard quantitative CFT scores have been widely used as clinical measures of SM, they are also significantly influenced by other, non-SM abilities (Aita et al., 2019; Elgamal et al., 2011; Gibbons et al., 2012; Rosen & Engle, 1997; Shao et al., 2014; Whiteside et al., 2016), which limits their potential to detect subtle SM decline. It is based on this limitation that item-level scores started receiving the attention of clinical researchers (Binetti et al., 1995; Rosen, 1980. In her manuscript, Rosen refers to the “clearest cases” to indicate “the most frequently given members of the category”). In carrying out this systematic review, we tested the hypotheses whereby CFT item-level scores would be sensitive to neurodegenerative processes (first hypothesis). Moreover, as ageing is associated with the continued acquisition of semantic knowledge, we also hypothesised that better item-level scores would be recorded among older adults when compared with younger adults (second hypothesis).

The studies included in this systematic review indicate that individuals who are along the clinical continuum between MCI and AD dementia generate words that tend to be semantically easier than those generated by healthy adults. This emerges from the largest majority of studies, in relation to at least one of the item-level features scored by the methodology. Frequency has been, by far, the feature most often investigated. Eight out of twelve cross-sectional studies reported a significant frequency-related impoverishment of CFT words in the MCI-AD clinical continuum (Binetti et al., 1995; Ferrante et al., 2024; Forbes-McKay et al., 2005; Marczinski & Kertesz, 2006; Paek & Murray, 2021; Pakhomov et al., 2016; Sailor et al., 2011; Won et al., 2021), while the only two longitudinal studies so far published confirm frequency as predictor of longitudinal outcomes in this clinical continuum (Pakhomov et al., 2016; Vonk et al., 2023). Age of acquisition has been the second most commonly studied feature. Seven out of eight cross-sectional studies indicate age-of-acquisition-related impoverishment in this same diagnostic continuum (Forbes-McKay et al., 2005; Paek & Murray, 2021; Rofes et al., 2020; Sailor et al., 2011; Venneri et al., 2008; Wakefield et al., 2018; Won et al., 2021). In addition, this feature was also reported as a significant predictor of diagnostic trajectories in the only longitudinal design that has included it (Vonk et al., 2023). As frequency and age of acquisition are simple constructs that could be potentially implemented in clinical settings, we tested their cross-sectional trends across studies via meta-analytical procedures, which confirmed the significant difference. Overall, these findings provide support to our first hypothesis.

Two moderators played a significant role in the meta-analysis of words’ age of acquisition. The number of CFT categories (i.e. 1, 2, or 3) was positively associated with the size of the effect. The use of multiple categories appears to “amplify” the difference between controls and patients, as the former can generate a larger number of words acquired later in life, while the latter cannot. Conversely, frequency was unaffected by the number of categories, suggesting a stable, rather than cumulative advantage in controls in relation to this feature. The size of the effect was also strongly associated with the educational level of MCI/AD patients. Educational attainment is one of the core proxies of cognitive reserve (Stern et al., 2020) and is also one of the best-established factors that protect against AD (Hersi et al., 2017). Higher levels of cognitive reserve might help preserve the qualitative aspects of the CFT performance of patients, and this would be particularly visible in relation to words’ age-of-acquisition as longer educational attainments result in people acquiring a larger number of words. This does not apply to words’ frequency, as normative data are typically collected via the analysis of a large corpus of linguistic data (e.g. van Heuven et al., 2014), and this is unrelated to educational attainment.

Four studies based on CFT item-level features have been carried out in individuals with a diagnosis of PD. These indicate a general decline in SM performance in this clinical group (which is also in support of our first hypothesis), but effects were also influenced by medication status, with levels of performance reported as normal in two out of three studies when patients were regularly on medication (Herrera et al., 2012; Zabberoni et al., 2017). Interestingly, the study by Herrera and colleagues (2012) indicated a selective difficulty shown by this clinical group (when OFF medication) in generating infrequent “action words”. This category embeds much more motor semantics than the more commonly used categories (such as “animals”) and, for this reason, is thought to be particularly sensitive to disruption of fronto-basal circuits (Woods et al., 2005). More studies are necessary to characterise the motor aspect of fluency words, both in relation to “motor categories” as well as motor semantics (Lynott et al., 2020) of “regular” categories. A methodological aspect that emerges from this consideration is the choice of categories, as two more studies carried out in MCI-AD participants reported effects limited to some but not all categories (Hough & Givens, 2004; Sailor et al., 2004). Categories are typically selected arbitrarily, with “animals”, “fruits”, and “vegetables” being, by far, those used most frequently. More research is needed to understand to what extent individual categories are interchangeable and allow for test–retest reliability.

Overall, the evidence of an effect of PD on item-level CFT is not as convincing as that emerging from the study of MCI and AD. All four investigations were carried out in individuals with no cognitive impairment who had normal quantitative CFT scores when ON medication. Semantic processing is supported by a wide network of cortical regions (Binder et al., 2009; Huth et al., 2016), while the early stage of mild PD affects the cortex only to a limited extent (Filippi et al., 2020). Since early-stage AD has a much more pronounced effect on the cortex, it is normal to expect worse item-level scores in this diagnosis. Moreover, studies carried out in PD report effects that are associated with clinical presentation (i.e. left-sided vs. right-sided symptoms), clinical management (i.e. individuals ON vs. OFF medication), and test methodology (i.e. CFT performance half and CFT category type), indicating a degree of selectivity in how PD affects item-level CFT scores (as opposed to a much more general effect seen in MCI and AD). In conclusion, more studies are needed to characterise item-level CFT performance in PD at its various clinical stages, including individuals with PD-MCI and PD-dementia.

Six studies investigated item-level features of CFT production in samples of individuals with a diagnosis of PPA or other form of neurodegeneration. While one of the studies focussed on diagnostic classification (Rofes et al., 2019), the other three indicated that individuals with a semantic variant of PPA had the poorest performance levels when compared with groups of individuals suffering from other forms of PPA or other neurodegeneration (Henderson et al., 2023; Marczinski & Kertesz, 2006; Van den Berg et al., 2024), although this was reported in a range of distinct features. Overall, these findings are in further support of our first hypothesis, but it is also fair to recognise that the evidence on bvFTD is more ambiguous, as one study reported impoverished item-level performance in this group compared with controls (Van den Berg et al., 2024), while other two studies did not find any effect in this group (Ferrante et al., 2024; Henderson et al., 2023). The study by Jiskoot and colleagues (2023), finally, suggests that genetic variability might contribute to semantic profiles in bvFTD and nfPPA.

The findings emerging from the study of normal ageing, conversely, do not seem to indicate any clear-cut trends. One study reported that older adults generated more single-occurrence words than young adults (Kavé et al., 2009), while a second study reported higher-occurrence scores in older adults than in young adults (Castro et al., 2021). Other studies reported no age-related differences in average word frequency or typicality (Hough, 2007; Vita et al., 2014), while two further studies reported instead a positive association between increasing age and average frequency (Murphy & Castel, 2021; Taler et al., 2020). Two studies, finally, investigated the link between the serial order (or position) of recall and item-level features, reporting differences between young and older adults in recall organisation according to relative occurrence (Murphy & Castel, 2021) and valence (De Marco et al., 2021). It is possible that ageing might influence some (but not all) item-level features, but the current collective evidence is not conclusive. In summary, these data do offer support to our second hypothesis and indicate that ageing does not have an effect on item-level CFT performance comparable to that of neurodegenerative conditions. Finally, two studies specifically tested the effect of the apolipoprotein ɛ4 allele on item-level CFT performance. While the presence of the ɛ4 allele is associated with significantly more frequent words in healthy older adults (Vonk et al., 2019b), no difference was reported in typicality and age of acquisition at the MCI stage (Venneri et al., 2011).

Other than to the CFT, item-level scores have been fruitfully applied also to other neuropsychological tests, such as the Letter Fluency Test (Foley et al., 2021), the Rey-Osterrieth Complex Figure (Salvadori et al., 2019), the Boston Naming Test (De Marco et al., 2023b), and the Prose Memory Test (Mueller et al., 2023), suggesting that the cognitive effort at the basis of each individual test item can be informative beyond summative scores. Ideally, to analyse the added value of item-level scores in characterising normal and abnormal ageing, standard quantitative scores should be used as correction factors in statistical models. Of the publications reviewed in the “Results” section, however, only five studies included quantitative scores as covariates in the relevant analyses (De Marco et al., 2021; van den Berg et al., 2024; Vita et al., 2014; Vonk et al., 2019b, 2023). As a result, while the literature on the topic does appear to support the study of item-level scores, future studies should provide more robust statistical control and identify the degree to which item-level scores are genuinely independent of quantitative scores.

Another element that is apparent from the review is the scarcity of studies, i.e. only that by Ferrante et al. (2024), that have adhered to the recent research diagnostic criteria of Alzheimer’s disease (Dubois et al., 2014; Jack et al., 2018). While diagnostic criteria for PD and PPA are better consolidated in the clinical practice (Gorno-Tempini et al., 2011; Postuma et al., 2015), diagnostic criteria for AD at the MCI and dementia stages have been shifting, over the last decade, from a clinical to a biological framework. In this respect, it still needs to be established whether item-level features of CFT performance are associated with the pathological processes of AD. Evidence from studies that recruited and followed up cohorts of adults, healthy at baseline, indicates that SM decline (measured with quantitative fluency scores) is visible at least six years before a diagnosis of AD is made (Amieva et al., 2008; Hirni et al., 2016; Payton et al., 2020), suggesting a link between this function and early-stage neuropathological changes. On this note, meta-analyses indicate that, although quantitative CFT scores are significant predictors of amyloid burden (Vonk et al., 2020), their link with TAU burden is non-significant (Pelgrim et al., 2021). This is despite the fact that evidence indicates that CFT scores are significantly associated with neuroradiological properties of the region distinctively affected by neurofibrillary tangles and neuropil threads during Braak Stages I and II, namely the perirhinal cortex (Hirni et al., 2013; Venneri et al., 2019), and a consolidated framework exists in support of a link between SM and the anterior portion of the parahippocampal gyrus where the perirhinal cortex is located (Mishkin et al., 1997). A possible explanation for such incongruency may reside in the construct validity of standard CFT scores, since, as pointed out in the “Introduction” section, performance on this test is also supported by other, “non-SM” abilities such as working memory, attention, and speed-of-processing. On this note, there is well-established evidence of neurological compensatory mechanisms (i.e. with particular evidence on those supported by the prefrontal lobe) playing a major role in supporting cognitive performance in ageing (Park & Reuter-Lorenz, 2009), suggesting that these may contribute to group variability in CFT performance. This goes hand in hand with the evidence that neurocognitive ageing follows a trajectory that varies across individuals (Lindenberger, 2014; Raz et al., 2010). As a result, the link between AD pathology and CFT performance is inevitably influenced by the inter-individual degree of reliance on extra-SM resources. This further indicates that studies are needed in order to understand the link between item-level scores and global and regional levels of pathology.

The evidence emerging from this systematic review indicates that item-level scoring of CFT performance may help characterise the clinical profile of individuals with a neurological diagnosis beyond the information provided by quantitative scores. This is confirmed by the meta-analysis of words’ frequency and age of acquisition carried out in patients with a clinical diagnosis of MCI or AD. It is possible, however, that mathematical solutions other than the simple calculation of average values might be better options to quantify the complexity of the words retrieved during the course of the CFT minute, such as the average of the first few words (Forbes-McKay et al., 2005; Sailor et al., 2004; Wakefield et al., 2018) or of most complex words (Vonk et al., 2023), or the measurement of the longitudinal trends of word complexity during CFT performance (De Marco et al., 2021; Murphy & Castel et al., 2021). Combinatory methods such as the use of graph theory (De Marco et al., 2021) or classification methods (Rofes et al., 2019, 2020) deserve further study as they can help quantify multi-dimensional aspects of semantic complexity that are not captured by regular univariate analyses. Moreover, it has also to be pointed out that the scoring and use of item-level methods should be adequately and fruitfully transposed to clinical settings (and to settings where the study of healthy ageing is central). At this stage, the route to extra-academic translation has not been yet appropriately addressed, although frequency and age of acquisition could be two candidates of interest.

In conclusion, although the literature on item-level scoring in normal and neurologically abnormal ageing is quite diverse, the resulting trend indicates that this method offers the opportunity to enrich the information provided by the CFT. Item-level scores contribute to defining a landscape of “non-conventional” CFT scoring methods that can be very useful in academic and clinical research. This arsenal of methodologies also includes the identification of clusters and switches (Troyer, 2000), the definition of Category Fluency-Letter Fluency differential scores (Marra et al., 2021; Wright et al., 2023), the analysis of CFT perseverations and intrusions (Perez et al., 2020), and the computation of lexical-semantic networks (Bertola et al., 2014; Sinha et al., 2022). This systematic review focused neither on Letter Fluency performance nor on scores indicative of clustering and switching (and this could be acknowledged as a limitation). Future systematic reviews should focus on these methodologies to expand the literature on the topic. All these approaches are theory-driven and entirely based on post-processing methodologies, which make them inexpensive and sensitive to aspects of performance that would otherwise be ignored.

## Supplementary Information

Below is the link to the electronic supplementary material.

Supplementary file1 (DOCX 32.2 KB)

## Data Availability

All data supporting the findings of this study are available within the paper and its Supplementary Information. Tables S1–S3 include all data used in the meta-analytical section of the study.
