# Supplementary material for: Item-Level Analysis of Category Fluency Test Performance: A Systematic Review and Meta-Analysis of Studies of Normal and Neurologically Abnormal Ageing
Source: Neuropsychol Rev. 2025 Jan 22;35(4):644–87. doi: 10.1007/s11065-024-09657-z (PMC12920347; doi:10.1007/s11065-024-09657-z)
Supplement: Supplementary file 1 — Supplementary file1 (DOCX 32.2 KB) [file 11065_2024_9657_MOESM1_ESM.docx]

**Supplementary Information**

1. Selected items from the study quality checklist by Downs and Black (1998) used in this systematic review

Reporting

**Q1**. Is the hypothesis/aim/objective of the study clearly described?

[YES = 1; NO = 0]

**Q2**. Are the main outcomes to be measured clearly described in the Introduction or Methods section?

[YES = 1; NO = 0]

*If the main outcomes are first mentioned in the Results section, the question should be answered NO.*

**Q3**. Are the characteristics of the patients included in the study clearly described?

[YES = 1; NO = 0]

*In cohort studies and trials, inclusion and/or exclusion criteria should be given. In case-control studies, a case-definition and the source for controls should be given.*

**Q5**. Are the distributions of principal confounders in each group of subjects to be compared clearly described?

[YES = 1; PARTIALLY = 1; NO = 0]

*A list of principal confounders is provided.*

**Q6**. Are the main findings of the study clearly described?

[YES = 1; NO = 0]

*Simple outcome data (including denominators and numerators) should be reported for all major findings so that the reader can check the major analyses and conclusions. (This question does not cover statistical tests which are considered below).*

**Q7**. Does the study provide estimates of the random variability in the data for the main outcomes?

[YES = 1; NO = 0]

*In non-normally distributed data the inter-quartile range of results should be reported. In normally distributed data the standard error, standard deviation or confidence intervals should be reported. If the distribution of the data is not described, it must be assumed that the estimates used were appropriate and the question should be answered YES.*

**Q10**. Have actual probability values been reported (e.g. 0.035 rather than <0.05) for the main outcomes except where the probability value is less than 0.001?

[YES = 1; NO = 0]

External Validity

**Q11**. Were the subjects asked to participate in the study representative of the entire population from which they were recruited?

[YES = 1; NO = 0; UNABLE TO DETERMINE = 0]

*The study must identify the source population for patients and describe how the patients were selected. Patients would be representative if they comprised the entire source population, an unselected sample of consecutive patients, or a random sample. Random sampling is only feasible where a list of all members of the relevant population exists. Where a study does not report the proportion of the source population from which the patients are derived, the question should be answered as UNABLE TO DETERMINE.*

**Q12**. Were those subjects who were prepared to participate representative of the entire population from which they were recruited?

[YES = 1; NO = 0; UNABLE TO DETERMINE = 0]

*The proportion of those asked who agreed should be stated. Validation that the sample was representative would include demonstrating that the distribution of the main confounding factors was the same in the study sample and the source population.*

Internal Validity

**Q16**. If any of the results of the study were based on “data dredging”, was this made clear?

[YES = 1; NO = 0; UNABLE TO DETERMINE = 0]

*Any analyses that had not been planned at the outset of the study should be clearly indicated. If no retrospective unplanned subgroup analyses were reported, then answer YES.*

**Q17**. In trials and cohort studies, do the analyses adjust for different lengths of follow-up of patients, or in case-control studies, is the time period between the intervention and outcome the same for cases and controls?

[YES = 1; NO = 0; UNABLE TO DETERMINE = 0]

*Where follow-up was the same for all study patients the answer should be YES. If different lengths of follow-up were adjusted for by, for example, survival analysis the answer should be YES. Studies where differences in follow-up are ignored should be answered NO.*

**Q18**. Were the statistical tests used to assess the main outcomes appropriate?

[YES = 1; NO = 0; UNABLE TO DETERMINE = 0]

*The statistical techniques used must be appropriate to the data. For example, nonparametric methods should be used for small sample sizes. Where little statistical analysis has been undertaken but where there is no evidence of bias, the question should be answered YES. If the distribution of the data (normal or not) is not described it must be assumed that the estimates used were appropriate and the question should be answered YES.*

**Q20**. Were the main outcome measures used accurate (valid and reliable)?

[YES = 1; NO = 0; UNABLE TO DETERMINE = 0]

*For studies where the outcome measures are clearly described, the question should be answered YES. For studies which refer to other work or that demonstrates the outcome measures are accurate, the question should be answered as YES.*

**Q21**. Were the patients in different intervention groups (trials and cohort studies) or were the cases and controls (case-control studies) recruited from the same population?

[YES = 1; NO = 0; UNABLE TO DETERMINE = 0]

*For example, patients for all comparison groups should be selected from the same hospital. The question should be answered UNABLE TO DETERMINE for cohort and case-control studies where there is no information concerning the source of patients included in the study.*

**Q22**. Were study subjects in different intervention groups (trials and cohort studies) or were the cases and controls (case-control studies) recruited over the same period of time?

[YES = 1; NO = 0; UNABLE TO DETERMINE = 0]

*For a study which does not specify the time period over which patients were recruited, the question should be answered as UNABLE TO DETERMINE.*

**Q25**. Was there adequate adjustment for confounding in the analyses from which the main findings were drawn?

[YES = 1; NO = 0; UNABLE TO DETERMINE = 0]

*In nonrandomised studies if the effect of the main confounders was not investigated or confounding was demonstrated but no adjustment was made in the final analyses the question should be answered as NO.*

1. Data selection and homogenisation procedures for meta-analysis - Frequency

Twelve studies (i.e., reported in **Fig. 3** and in the first part of **Section 3.2.1.1**) scored words’ *frequency* and analysed the effect of the MCI-to-AD clinical continuum via patient-vs.-control between-group comparison models. **Tables S1-S2** includes the effects used as part of the meta-analysis and all moderators.

**Table S1**. Studies included in the meta-analysis of frequency data relying on sample mean, SD and size for the calculation of the effects

| **Study** | **OUTCOME** | | | | | | **MODERATORS** | | | | | |
| --- | --- | --- | --- | --- | --- | --- | --- | --- | --- | --- | --- | --- |
|  | **AD/MCI individuals** | | | **Controls** | | | **Quality Rating** | **AD/MCI group average** | | | **CFT categories** | **CFT count difference** |
|  | **Frequency (*mean*)** | **Frequency (*SD*)** | ***n*** | **Frequency (*mean*)** | **Frequency (*SD*)** | ***n*** |  | **Age** | **Education** | **MMSE** |  |  |
| Binetti et al., 1995 | 6.7 | 4.1 | 40 | 4.4 | 2.4 | 35 | 62.50% | 69.7 | 7.2 | 21.4 | 1 | 5.8 |
| Forbes McKay et al., 2005 | 16.63 | 6.51 | 34 | 11.9 | 3.08 | 40 | 62.50% | 76.65 ^a^ | 10.55 ^a^ | 21.84 | 2 | 14.64 |
| Henderson et al., 2023 | 4.26 | 0.26 | 18 | 3.93 | 0.16 | 33 | 60.00% | 67 | 12 ^b^ | 21 | 1 | 13.85 |
| Paek and Murray, 2021 | 3.761 | 0.263 | 11 | 3.496 | 0.285 | 12 | 68.75% | 75.83 | 15.5 | 23.5 | 1 | 3.92 |
| Pakhomov et al., 2016 | 10.256 ^e^ | 1.823 ^e^ | 71 | 10.087 ^e^ | 1.484 ^e^ | 46 | 81.25% | N/A | N/A | N/A | 1 | 4.685 ^e^ |
| Sailor et al., 2011 | 12.5 | 0.43 | 22 | 11.9 | 0.83 | 34 | 75.00% | 77.9 | 10.9 | 20.18 ^c^ | 3 | 16 |
| Venneri et al., 2008 | 8.39 | 2.81 | 25 | 8.43 | 2.29 | 25 | 56.25% | 75.88 | 11.72 | 22.96 | 2 | 19.66 |
| Vita et al., 2014 | 9.89 | 3.325 | 60 | 8.49 | 2.354 | 20 | 76.47% | 71.33 | 10.27 | 25.98 | 2 | 4.4 |
| Won et al., 2021 | 2.8 | 0.3 | 17 | 2.6 | 0.2 | 18 | 62.50% | 79.5 | 15.6 | 24.9 ^d^ | 1 | 7.9 |

^a.^ The publication included three groups of individuals across the MCI-to-AD continuum of various severity. The three values included in the table, however, reflect the average across the entire cohort (i.e., of *n* = 96);

^b.^ The publication included “mean age at leaving full-time education (years)”, which were converted to education years based on the age at which individuals start going to school in the UK (i.e., at the age of 5);

^c.^ The publication included Blessed Information-Memory-Concentration (BIMC) test scores as indices of clinical severity. The formula published by Thal and colleagues (1986) was used to convert the score of 10.9 reported by the authors to a MMSE equivalent;

^d.^ Clinical severity was reported in a different publication (i.e., Carson-Smith et al., 2015), and only in relation to 9 of the 17 individuals included in the study;

^e.^ The average count of words generated by each group, the average frequency value, and the average standard error score (subsequently converted into standard deviation) were estimated from the figure included in the publication;

AD: Alzheimer’s disease; CFT: category fluency test; MCI: mild cognitive impairment; MMSE: Mini Mental State Examination; SD: standard deviation

**Table S2**. Studies included in the meta-analysis of frequency data relying on sample size and *p*-values to calculate effects

| **Study** | **OUTCOME** | | | **MODERATORS** | | | | | |
| --- | --- | --- | --- | --- | --- | --- | --- | --- | --- |
|  | ***p*-value** | ***n* (AD/MCI individuals)** | ***n* (Controls)** | **Quality Rating** | **AD/MCI group average** | | | | |
|  |  |  |  |  | **Age** | **Education** | **MMSE** | **CFT categories** | **CFT count difference** |
| Beber et al., 2015 | 0.762 ^e^ | 35 | 35 | 62.50% | 73.14 | 4.37 | 16.6 | 1 | 4.16 |
| Ferrante et al., 2023 | 0.03 ^f^ | 32 | 27 | 87.50% | 75.75 | 11.63 | 21 ^b^ | 1 | 10.1 |
| Marczinski and Kertesz, 2006 | 0.05 ^f^ | 20 | 20 | 75.00% | 74.7 | 9.9 | 21.3 | 2 | 7.3 |

^a.^ The publication includes an *F*-statistic and a *p*-value, as the inferential model was designed to compare three, not two groups. Given the non-significance of the result, however, the study was included in the meta-analysis following a conservative approach;

^b.^ The *p*-value was associated with a *post-hoc* Tukey’s Honestly Significant Difference test;

^c.^ The *p*-value was associated with a *post-hoc* Fisher’s Least Significant Difference test and was reported as “< .05”;

^d.^ The *p*-value was only reported in an image only, and marked as “**”;

^e.^ The publication included MOntreal Cognitive Assessment (MoCA) test scores as indices of clinical severity. The grid published by Fasnacht and colleagues (2023) was used to convert the rounded down score of 15 (originally 15.41) to an MMSE equivalent;

^f.^ The average count of words generated on average by each group was estimated from the figure included in the publication;

AD: Alzheimer’s disease; CFT: category fluency test; MCI: mild cognitive impairment; MMSE: Mini Mental State Examination; SD: standard deviation

1. Data selection and homogenisation procedures for meta-analysis – Age of acquisition

Seven studies (i.e., reported in **Fig. 3**) scored words’ *age of acquisition* and analysed the effect of the MCI-to-AD clinical continuum via patient-vs.-control between-group comparison models. An eight one (Venneri et al., 2011) was also included: this publication reported age-of-acquisition scores in controls and two groups of MCI individuals, i.e., APOE ɛ_4_ carriers and non-carriers. The latter was included in the meta-analysis. **Table S3** reports the effects used as part of the meta-analyses and all moderators.

**Table S3**. Studies included in the meta-analysis of age-of-acquisition data (i.e., all relying on sample mean, SD and size for the calculation of the effects)

| **Study** | **OUTCOME** | | | | | |  | **MODERATORS** | | | | |
| --- | --- | --- | --- | --- | --- | --- | --- | --- | --- | --- | --- | --- |
|  | **AD/MCI individuals** | | | **Controls** | | | **Quality Rating** | **AD/MCI group average** | | | **CFT Categories** | **CFT count difference** |
|  | **Age of acquisition (*mean*)** | **Age of acquisition (*SD*)** | ***n*** | **Age of acquisition (*mean*)** | **Age of acquisition (*SD*)** | ***n*** |  | **Age** | **Education** | **MMSE** |  |  |
| Forbes McKay et al., 2005 | 4.96 | 0.82 | 34 | 6.27 | 0.62 | 40 | 62.50% | 76.65 ^a^ | 10.55 ^a^ | 21.84 ^a^ | 2 | 20.05 |
| Henderson et al., 2023 | 2.29 | 0.27 | 18 | 2.62 | 0.21 | 33 | 60.00% | 67 | 12 ^b^ | 21 | 1 | 13.85 |
| Paek and Murray, 2021 | 4.531 | 0.305 | 11 | 5.104 | 0.737 | 12 | 68.75% | 75.83 | 15.5 | 23.5 | 1 | 3.92 |
| Sailor et al., 2011 | 5.491 ^d^ | 0.326 ^d^ | 22 | 6.098 ^d^ | 0.343 ^d^ | 34 | 75.00% | 77.9 | 10.9 | 20.18 ^c^ | 3 | 16 |
| Venneri et al., 2008 | 4.96 | 0.85 | 25 | 6.32 | 0.73 | 25 | 56.25% | 75.88 | 11.72 | 22.96 | 2 | 19.6 |
| Venneri et al., 2011 | 4.84 | 0.56 | 14 | 6.53 | 0.96 | 11 | 68.75% | 67.78 | 7.57 | 27.07 | 2 | 12.97 |
| Wakefield et al., 2018 | 4.88 | 0.403 | 20 | 5.51 | 0.403 | 20 | 75.00% | 66.25 | 11 | 26.4 | 2 | 22.05 |
| Won et al., 2021 | 5 | 0.7 | 17 | 5.5 | 0.6 | 18 | 62.50% | 79.5 | 15.6 | 24.9 ^e^ | 1 | 7.9 |

^a.^ The publication included three groups of individuals across the MCI-to-AD continuum of various severity. The three values included in the table, however, reflect the average across the entire cohort (i.e., of *n* = 96);

^b.^ The publication included “mean age at leaving full-time education (years)”, which were converted to education years based on the age at which individuals start going to school in the UK (i.e., at the age of 5);

^c.^ The publication included Blessed Information-Memory-Concentration (BIMC) test scores as indices of clinical severity. The formula published by Thal and colleagues (1986) was used to convert the score of 10.9 reported by the authors to a MMSE equivalent;

^d.^ The publication reports age of acquisition values (i.e., mean and SD) on a 7-point scale multiplied by 100. The values included in the meta-analysis were calculated via a set of proportions;

^e.^ Clinical severity was reported in a different publication (i.e., Carson-Smith et al., 2015), and only in relation to 9 of the 17 individuals included in the study;

AD: Alzheimer’s disease; CFT: category fluency test; MCI: mild cognitive impairment; MMSE: Mini Mental State Examination; SD: standard deviation

**Additional References** (i.e., not included in the main manuscript)

Carson Smith, J., Nielson, K. A., Antuono, P., Lyons, J. A., Hanson, R. J., Butts, A. M., Hantke, N. C., & Verber, M. D. (2013). Semantic memory fMRI and cognitive function after exercise intervention in mild cognitive impairment. Journal of Alzheimer’s Disease, 37(1), 197–215. <https://doi.org/10.3233/JAD-130467>

Fasnacht, J. S., Wueest, A. S., Berres, M., Thomann, A. E., Krumm, S., Gutbrod, K., Steiner, L. A., Goettel, N., & Monsch, A. U. (2023). Conversion between the montreal cognitive assessment and the mini-mental status examination. Journal of the American Geriatrics Society, 71(3), 869–879. <https://doi.org/10.111/jgs.18124>

Thal, L. J., Grundman, M., & Golden, R. (1986). A correlational analysis of the Blessed Information-Memory-Concentration test and the mini-mental state exam. Neurology, 36(2), 262–264. <https://doi.org/10.1212/wnl.36.2.26>
